# Supplementary material for: Neonatal mortality in NHS maternity units by timing and mode of birth: a retrospective linked cohort study
Source: BMJ Open. 2023 Jun 12;13(6):e067630. doi: 10.1136/bmjopen-2022-067630 (PMC10277044; doi:10.1136/bmjopen-2022-067630)
Supplement: Supplementary data [file bmjopen-2022-067630supp001.pdf]

Neonatal mortality in NHS maternity units by timing and method of birth: a retrospective linked cohort study

Lucy Carty, Christopher Grollman, Rachel Plachcinski, Mario Cortina-Borja and Alison Macfarlane

[Citation detail]

DOI: [Article DOI]

Supplementary material

Coding of all variables used in the analysis

Coding of variables used in the analysis.

Supplementary table 1. Coding of all variables used in the analysis

Variable in analysis	Original format	Coding
Baby's date of birth	Calendar date	Continuous from first to last day of study
Day in year of baby's date of birth	Calendar date	Continuous from 1 to 365 or 366
Clock go forward/back	Calendar date	Binary for whether clocks went forward/back that day
Working hours (binary)	Time of birth, calendar date	Binary:<br>Working hours = 0700 to 1859 inclusive, Monday to Friday except public holidays<br>Non-working hours = all other times
Working hours (4 categories)	Time of birth, calendar date	Four categories:<br>Working hours (weekday daytime) = 0700 to 1859, Monday to Friday except public holidays<br>Weekday night-time = 1900 to 2359, Monday to Friday except public holidays; 0000 to 0659 Tuesday to Friday except public holidays<br>Weekend/holiday daytime = 0700 to 1859 Saturday, Sunday or public holiday<br>Weekend/holiday night-time = 1900 to 2359, Saturday, Sunday or public holiday; 0000 to 0659 Saturday, Sunday, public holiday, Monday or day after public holiday
Gestational age	Completed weeks since last menstrual period, or by ultrasound	Grouped in categories:<br>22 to 28 weeks; 28 to 32 weeks; 32 to 37 weeks; 37 to 42 weeks; 42 weeks and over; Missing

Birthweight	Birthweight in grammes	Grouped in categories:<br><br><2500; 2500–2999; 3000–3499; 3500–3999; 4000+
Age of mother	Fractional years	Continuous in completed years
Parity	Number of previous births	Binary nulliparous/multiparous
Ethnicity	17 categories based on the ethnicity question in the 2001 Census of England and Wales	Seventeen categories:<br><br>White British; White Irish; Any other White background; Mixed White & Black Caribbean; Mixed White & Black African; Mixed White & Asian; Any other Mixed background; Asian Indian; Asian Pakistani; Asian Bangladeshi; Any other Asian background; Black Caribbean; Black African; Any other Black background; Chinese; Any other ethnic group; Not Known
Region	Ten Strategic Health Authority regions in the NHS for the majority of the study period	Ten categories: North East; North West; Yorkshire/Humber; East Midlands; West Midlands; East of England; London; South East Coast; South West; South Central
Marital status at birth registration	Categories recorded at birth registration	Four categories:<br><br>Joint registration with different address; Joint same address; Sole registration; Within marriage
Mother's place of birth	Countries recorded at birth registration	Grouped into seven categories:<br><br>United Kingdom; Other Europe; Africa; The Americas and the Caribbean; Middle East and Asia; Oceania and Antarctica; Missing
Previous caesarean birth	Whether present (yes/no)	Binary no/any previous caesarean birth
Obstetric complications*	Whether present (yes/no)	Binary not present/present

* Hypertension, Preeclampsia, Eclampsia, Diabetes, Gestational diabetes, Malpresentation of foetus, Maternal care for disproportion, Maternal care for abnormality of pelvic organs, Polyhydramnios, Oligohydramnios, Premature rupture of membranes, Placental disorders, Placenta praevia, Premature separation of placenta, Antepartum haemorrhage, Obstructed labour, Intrapartum haemorrhage, Umbilical cord complications

Crude all-cause neonatal mortality rates

Crude all-cause neonatal mortality rates in the analysis population were higher during non-working hours compared with working hours (1.58 vs 1.36 per 1000 live births, rate ratio 1.17, 95% confidence interval 1.12 to 1.22) (Supplementary table 2). The crude mortality rate ratio was not significantly different from 1 for spontaneous or instrumental births and was below 1 for emergency caesarean births (0.86, 0.80 to 0.93), meaning lower crude all-cause neonatal mortality rate during non-working hours compared with working hours (3.13 vs 3.62 per 1000 live births).

Supplementary table 2. Crude all-cause neonatal mortality by time and day of birth, stratified by method of birth

	Living at 28 days	Neonatal death	Total	Rate*	Rate ratio	95% CI
**Total population**						
Working hours	2325569	3162	2328731	1.36		
Non-working hours	3630159	5758	3635917	1.58	1.17	1.12 to 1.22
**Spontaneous delivery**						
Working hours	1201063	1647	1202710	1.37		
Non-working hours	2554815	3542	2558357	1.38	1.01	0.95 to 1.07
**Instrumental delivery**						
Working hours	256279	173	256452	0.67		
Non-working hours	485085	340	485425	0.70	1.04	0.87 to 1.25
**Emergency caesarean**						
Working hours	310092	1126	311218	3.62		
Non-working hours	553239	1735	554974	3.13	0.86	0.8 to 0.93
**Planned caesarean**						
Working hours	552917	200	553117	0.36		
Non-working hours	29791	82	29873	2.74	7.61	5.86 to 9.8

* Per 1000 live births

Adjusted odds of all-cause neonatal mortality by method of birth

After adjustment, the 'protective' effect of birth in non-working hours for emergency caesareans, seen in the crude rates, was no longer evident. Odds ratios for spontaneous, instrumental and emergency caesarean deliveries were not significantly different from 1 (Supplementary table 3).

Supplementary table 3. Unadjusted and adjusted odds ratios for all-cause neonatal mortality during non-working hours versus working hours by delivery type

	Odds ratio for neonatal mortality during non-working hours compared with working hours			
	Unadjusted OR	95% CI	Adjusted OR*	95% CI
**All-cause neonatal mortality**				
Spontaneous delivery	1.01	0.95 to 1.07	0.99	0.93 to 1.06
Instrumental delivery	1.04	0.87 to 1.25	1.11	0.91 to 1.34
Emergency caesarean	0.86	0.80 to 0.93	1.06	0.98 to 1.14

* Adjusted for baby's sex, gestational age, birthweight, mode of onset of labour, geographical region, NHS trust, baby's ethnicity, baby's date of birth, maternal age and maternal parity, as well as yearly harmonic terms for day of year of the birth.

Carty L, *et al. BMJ Open* 2023; 13:e067630. doi: 10.1136/bmjopen-2022-067630

Obstetric risk factor characteristics for emergency caesareans without labour

We compared obstetric risk factor characteristics for emergency caesareans without labour during working and non-working hours (supplementary materials) (Supplementary table 4).

Supplementary table 4. Obstetric characteristics and risk factors for emergency caesareans without labour by working and non-working hours

Characteristics and risk factors	Level	Working hours	%	Non-working hours	%	*p*-value	SMD
N		60,202		71,700			
Baby's sex	F	28,505	47.3	33,670	47.0	0.160	0.008
	M	31,697	52.7	38,030	53.0		
Gestational age	22 to 28 weeks	1,127	1.9	1,439	2.0	<0.001	0.034
	28 to 32 weeks	4,182	6.9	4,926	6.9		
	32 to 37 weeks	14,368	23.9	17,167	23.9		
	37 to 42 weeks	38,228	63.5	45,008	62.8		
	42+ weeks	2,062	3.4	2,880	4.0		
	Missing	235	0.4	280	0.4		
Birthweight	Under 2,500	16,653	27.7	20,629	28.8	<0.001	0.054
	2,500-2,999	11,214	18.6	14,367	20.0		
	3,000-3,499	15,229	25.3	17,638	24.6		
	3,500-3,999	10,759	17.9	12,083	16.9		
	4,000 and over	5,156	8.6	5,691	7.9		
	Missing or unfeasible	1,191	2.0	1,292	1.8		
Maternal age	Under 20	1,899	3.2	2,697	3.8	<0.001	0.060
	20-24	8,229	13.7	10,529	14.7		
	25-29	14,603	24.3	18,051	25.2		
	30-34	18,359	30.5	21,420	29.9		
	35-39	12,920	21.5	14,444	20.1		
	Over 40	4,192	7.0	4,559	6.4		
Parity	Nulliparous	22,316	37.1	29,730	41.5	<0.001	0.091
	Moderate: 1 to 4	35,249	8.6	38,885	54.2		
	High: 5 to 9	2,511	4.2	2,940	4.1		
	Very high: 10 to 14	111	0.2	132	0.2		
	Extreme, unfeasible or missing	X	0.0	X	0.0		
Ethnicity	White British	35,770	59.4	41,519	57.9	<0.001	0.041
	White Irish	427	0.7	537	0.7		
	Any other White background	3,946	6.6	4,714	6.6		
	Mixed White & Black Caribbean	581	1.0	765	1.1		

Carty L, *et al. BMJ Open* 2023; 13:e067630. doi: 10.1136/bmjopen-2022-067630

	Mixed White & Black African	427	0.7	491	0.7		
	Mixed White & Asian	670	1.1	814	1.1		
	Any other Mixed background	1,064	1.8	1,365	1.9		
	Asian Indian	2,349	3.9	3,056	4.3		
	Asian Pakistani	2,910	4.8	3,706	5.2		
	Asian Bangladeshi	1,157	1.9	1,500	2.1		
	Any other Asian background	1,171	1.9	1,352	1.9		
	Black Caribbean	772	1.3	991	1.4		
	Black African	3,377	5.6	4,262	5.9		
	Any other Black background	609	1.0	737	1.0		
	Chinese	239	0.4	275	0.4		
	Any other ethnic group	1,378	2.3	1,698	2.4		
	Not Known	3,355	5.6	3,918	5.5		
Region	North East	2,053	3.4	2,405	3.4	<0.001	0.080
	North West	10,040	16.7	12,628	17.6		
	Yorkshire/Humber	4,509	7.5	5,325	7.4		
	East Midlands	2,974	4.9	3,082	4.3		
	West Midlands	8,753	14.5	12,053	16.8		
	East of England	6,423	10.7	7,572	10.6		
	London	12,590	20.9	14,129	19.7		
	South East Coast	4,254	7.1	5,047	7.0		
	South West	3,589	6	4,059	5.7		
	South Central	5,017	8.3	5,400	7.5		
Marital status at birth registration	Joint registration different address	5,524	9.2	7,082	9.9	<0.001	0.045
	Joint registration same address	16,280	27	20,222	28.2		
	Sole registration	3,749	6.2	4,702	6.6		
	Within marriage	34,649	57.6	39,694	55.4		
Mother's place of birth	United Kingdom	42,735	71	50,128	69.9	<0.001	0.028
	Other Europe	4,070	6.8	4,997	7.0		
	Africa	5,022	8.3	6,242	8.7		
	The Americas and the Caribbean	1,096	1.8	1,258	1.8		
	Asia and Middle East	6,955	11.6	8,733	12.2		
	Oceania, Antarctica and other	298	0.5	304	0.4		
	Missing	26	0.0	38	0.1		
Previous caesarean	Yes	19,030	31.6	18,119	25.3	<0.001	0.141

Hypertension	Yes	942	1.6	956	1.3	<0.001	0.019
Preeclampsia	Yes	6,902	11.5	8,391	11.7	0.181	0.007
Eclampsia	Yes	294	0.5	352	0.5	0.978	<0.001
Diabetes	Yes	1,600	2.7	1,463	2.0	<0.001	0.041
Gestational diabetes	Yes	3,333	5.5	3,401	4.7	<0.001	0.036
Malpresentation of foetus	Yes	9,605	16	10,639	14.8	<0.001	0.031
Maternal care for disproportion	Yes	104	0.2	155	0.2	0.087	0.010
Maternal care for abnormality of pelvic organs	Yes	17,269	28.7	16,217	22.6	<0.001	0.139
Polyhydramnios	Yes	1,301	2.2	1,377	1.9	0.002	0.017
Oligohydramnios	Yes	2,014	3.3	1,973	2.8	<0.001	0.035
Premature rupture of membranes	Yes	5,874	9.8	8,560	11.9	<0.001	0.070
Placental disorders	Yes	1,375	2.3	1,577	2.2	0.310	0.006
Placenta praevia	Yes	3,374	5.6	3,436	4.8	<0.001	0.037
Premature separation of placenta	Yes	1,831	3	3,305	4.6	<0.001	0.082
Antepartum haemorrhage	Yes	2,178	3.6	3,327	4.6	<0.001	0.051
Obstructed labour	Yes	3,280	5.4	4,502	6.3	<0.001	0.035
Intrapartum haemorrhage	Yes	410	0.7	570	0.8	0.018	0.013
Umbilical cord complications	Yes	1,166	1.9	1,864	2.6	<0.001	0.045

SMD = standardised mean difference; X = suppressed due to count(s) below 10

Point estimates from final fully adjusted model

Supplementary table 5. Odds ratios for neonatal mortality attributed to anoxia, asphyxia or trauma among emergency caesarean births without labour from 2005 to 2014

	Odds ratio	95% CI lower	95% CI upper
Spline of baby's date of birth	1.415	0.586	3.416
Spline of baby's date of birth squared	0.611	0.386	0.967
Sine term related to day-in-year	0.994	0.833	1.185
Cosine term related to day-in-year	1.031	0.867	1.226
Clocks go forward*	0	NA	NA
Clocks go back*	0	NA	NA
Weekday daytime	Ref		
Weekday night-time	1.558	1.152	2.106
Weekend/holiday daytime	0.954	0.626	1.456
Weekend/holiday night-time	1.753	1.242	2.473

Gestational age 22 to 28 weeks	0.608	0.212	1.747
Gestational age 28 to 32 weeks	0.775	0.417	1.440
Gestational age 32 to 37 weeks	1.109	0.773	1.590
Gestational age 37 to 42 weeks	Ref		
Gestational age 42 weeks and over	0.348	0.128	0.948
Gestational age missing	0.825	0.113	6.034
Birth weight under 2,500	0.981	0.623	1.545
Birth weight 2,500 to 2,999	1.367	0.931	2.008
Birth weight 3,000 to 3,499	Ref		
Birth weight 3,500 to 3,999	1.136	0.744	1.733
Birth weight 4,000 and over	1.353	0.809	2.261
Birth weight missing or unfeasible	2.774	1.395	5.517
North East	1.349	0.613	2.967
North West	1.090	0.658	1.807
Yorkshire/Humber	2.132	1.244	3.653
East Midlands	1.812	0.884	3.716
West Midlands	1.343	0.804	2.244
East of England	1.322	0.761	2.295
London	Ref		
South East Coast	1.121	0.577	2.178
South Central	1.700	0.967	2.990
South West	1.619	0.880	2.978
Spline of age of mother	0.536	0.161	1.787
Spline of age of mother squared	0.175	0.022	1.371
Nulliparous	1.184	0.906	1.547
Multiparous	Ref		
Parity missing*	0	NA	NA
Placental abruption	4.526	3.279	6.248
Maternal treatment for pelvic abnormality	0.357	0.229	0.558
Malpresentation	0.279	0.155	0.500
Preeclampsia	0.306	0.160	0.585
Postpartum haemorrhage	1.704	1.287	2.257
Antepartum haemorrhage	2.282	1.547	3.367

* No deaths; Ref = reference category; CI = confidence interval

Neonatal deaths attributed to anoxia, asphyxia or trauma by year

The number of births each year by emergency caesarean without labour rose from 2005 to 2014, with a larger rise in births during non-working hours than in working hours. Applying the number needed to harm of 1258 to those births during weeknights and 933 to those births during weekend/holiday nights gives a number of annual deaths potentially associated with birth during non-working hours ranging from 3.2 deaths in 2005 to 6.2 deaths in 2014 (Supplementary table 6).

Supplementary table 6. Number of deaths per year attributable to anoxia, asphyxia or trauma potentially associated with birth during non-working hours, among emergency caesarean deliveries without labour

Carty L, *et al. BMJ Open* 2023; 13:e067630. doi: 10.1136/bmjopen-2022-067630

Birth year	Weekday daytime	Weekday night-time (NNH = 1258)		*Weekend/holiday daytime (NNH = 15,373)**		Weekend/holiday night-time (NNH = 933)		
	N births	N births	Excess deaths**	*N births*	*Excess deaths*	N births	Excess deaths	Total excess deaths
2005	4416	2317	1.8	*1435*	*0.1*	1252	1.3	3.2
2006	4650	2448	1.9	*1426*	*0.1*	1273	1.4	3.3
2007	4623	2378	1.9	*1382*	*0.1*	1223	1.3	3.2
2008	4716	2493	2.0	*1535*	*0.1*	1277	1.4	3.4
2009	6020	3505	2.8	*2020*	*0.1*	1873	2.0	4.8
2010	6450	3610	2.9	*2087*	*0.1*	1931	2.1	4.9
2011	6855	3920	3.1	*2385*	*0.2*	2159	2.3	5.4
2012	7336	4204	3.3	*2581*	*0.2*	2324	2.5	5.8
2013	7455	4200	3.3	*2509*	*0.2*	2330	2.5	5.8
2014	7681	4483	3.6	*2708*	*0.2*	2432	2.6	6.2
Total			26.7		NA		19.4	46.0

* Not included in total as the association of birth during weekend and holiday daytimes with neonatal mortality attributed to anoxia, asphyxia or trauma was not significant

** N births / NNH (number needed to harm)

Carty L, *et al. BMJ Open* 2023; 13:e067630. doi: 10.1136/bmjopen-2022-067630
