# Supplementary material for: Neonatal mortality in NHS maternity units by timing and mode of birth: a retrospective linked cohort study
Source: BMJ Open. 2023 Jun 12;13(6):e067630. doi: 10.1136/bmjopen-2022-067630 (PMC10277044; doi:10.1136/bmjopen-2022-067630)
Supplement: Author's manuscript [file bmjopen-2022-067630.draft_revisions.pdf]

BMJ Open

BMJ Open is committed to open peer review. As part of this commitment we make the peer review history of every article we publish publicly available.

When an article is published we post the peer reviewers' comments and the authors' responses online. We also post the versions of the paper that were used during peer review. These are the versions that the peer review comments apply to.

The versions of the paper that follow are the versions that were submitted during the peer review process. They are not the versions of record or the final published versions. They should not be cited or distributed as the published version of this manuscript.

BMJ Open is an open access journal and the full, final, typeset and author-corrected version of record of the manuscript is available on our site with no access controls, subscription charges or pay-per-view fees (http://bmjopen.bmj.com).

If you have any questions on BMJ Open's open peer review process please email info.bmjopen@bmj.com

BMJ Open

Neonatal mortality in NHS maternity units by timing and mode of birth: a retrospective linked cohort study

Journal:	*BMJ Open*
Manuscript ID	bmjopen-2022-067630
Article Type:	Original research
Date Submitted by the Author:	26-Aug-2022

Complete List of Authors:	Carty, Lucy; City University of London, Midwifery<br>Grollman, Christopher; City University of London, Midwifery<br>Plachcinski, Rachel; City University of London, Midwifery<br>Cortina Borja, Mario; University College London, UCL Institute of Child Health<br>Macfarlane, Alison; City University of London, Midwifery

Keywords:	NEONATOLOGY, OBSTETRICS, HEALTH SERVICES ADMINISTRATION & MANAGEMENT

SCHOLARONE™
Manuscripts

I, the Submitting Author has the right to grant and does grant on behalf of all authors of the Work (as defined in the below author licence), an exclusive licence and/or a non-exclusive licence for contributions from authors who are: i) UK Crown employees; ii) where BMJ has agreed a CC-BY licence shall apply, and/or iii) in accordance with the terms applicable for US Federal Government officers or employees acting as part of their official duties; on a worldwide, perpetual, irrevocable, royalty-free basis to BMJ Publishing Group Ltd ("BMJ") its licensees and where the relevant Journal is co-owned by BMJ to the co-owners of the Journal, to publish the Work in this journal and any other BMJ products and to exploit all rights, as set out in our licence.

The Submitting Author accepts and understands that any supply made under these terms is made by BMJ to the Submitting Author unless you are acting as an employee on behalf of your employer or a postgraduate student of an affiliated institution which is paying any applicable article publishing charge ("APC") for Open Access articles. Where the Submitting Author wishes to make the Work available on an Open Access basis (and intends to pay the relevant APC), the terms of reuse of such Open Access shall be governed by a Creative Commons licence – details of these licences and which Creative Commons licence will apply to this Work are set out in our licence referred to above.

Other than as permitted in any relevant BMJ Author's Self Archiving Policies, I confirm this Work has not been accepted for publication elsewhere, is not being considered for publication elsewhere and does not duplicate material already published. I confirm all authors consent to publication of this Work and authorise the granting of this licence.

For peer review only

Neonatal mortality in NHS maternity units by timing and mode of birth: a retrospective linked cohort study

Lucy Carty* (0000-0003-2849-3145)

Chris Grollman* (0000-0002-6950-1837)

Rachel Plachcinski (0000-0001-9908-0773)

Mario Cortina-Borja (0000-0003-0627-2624)

Alison Macfarlane (0000-0003-0977-7214)

* Joint first authors

Name	Affiliation	Email	ORCID
Lucy Carty	City, University of London	lucy.carty@city.ac.uk	0000-0003-2849-3145
Christopher Grollman	City, University of London	christopher.grollman@city.ac.uk	0000-0002-6950-1837
Mario Cortina-Borja	University College London	m.cortina@ucl.ac.uk	0000-0003-0627-2624
Rachel Plachcinski	City, University of London	rachel.plachcinski@city.ac.uk	0000-0001-9908-0773
Alison Macfarlane**	City, University of London	a.j.macfarlane@city.ac.uk	0000-0003-0977-7214

** Corresponding author

Word count for the main text (excluding the abstract, references, tables, boxes, or figures): 5678

Summary box

What is already known

- The 'weekend effect' in healthcare, whereby risks of poor outcomes may be higher outside standard working hours, has been a policy focus in England in the last decade.
- For perinatal outcomes including neonatal mortality, studies from high-income countries have shown varied and inconsistent evidence of worse outcomes 'out of hours'.
- Data from England have suggested higher perinatal mortality at the weekend, and data from Scotland have shown higher rates of death 'out of hours' ascribed to intrapartum anoxia but not to other causes.

What this study adds

- In the six million NHS births in England from 2005 to 2014, there was no association between timing of birth and neonatal mortality among spontaneous or instrumental births, or among emergency caesarean births following labour. These constitute 98% of births.
- For babies born by emergency caesarean without labour (2% of births), there was an increased risk of neonatal mortality by anoxia, asphyxia or trauma among those born at night compared to during the day. This translated to a slightly higher absolute risk.

For peer review only - http://bmjopen.bmj.com/site/about/guidelines.xhtml

Structured abstract

Objectives To compare neonatal mortality in English hospitals by time of day and day of the week according to care pathway.

Design Retrospective cohort linking birth registration, birth notification and hospital episode data

Setting NHS hospitals in England

Participants 6,054,536 liveborn singleton births from 2005 to 2014 in NHS maternity units in England

Main outcome measures Neonatal mortality

Results

After adjustment for confounders, there was no significant difference in the odds of neonatal mortality attributed to anoxia, asphyxia and trauma outside of working hours compared with working hours for spontaneous births or instrumental births. Stratification of emergency caesareans by onset of labour showed no difference in mortality by birth timing for emergency caesareans with spontaneous or induced onset of labour. Higher odds of neonatal mortality attributed to anoxia, asphyxia and trauma during non-working hours for emergency caesareans without labour translated to a small absolute difference in mortality risk.

Conclusions

The apparent 'weekend effect' may result from deaths among the relatively small numbers of babies who were coded as born by emergency caesarean section without labour outside normal working hours. Obstetric staffing should be planned to enable response to these relatively unusual emergencies.

Introduction

Since 2001, a considerable number of analyses have shown higher mortality rates among patients admitted to hospital at weekends compared with weekdays. There has been considerable debate about how to interpret these rates, with differences in quality of care and differences in case mix being frequently cited;[1, 2] concerns about a 'weekend effect' led to a '7-day services' policy for the NHS in England in 2015.[3] A systematic review looking at the need to increase specialist intensity at weekends for patients undergoing emergency hospital admission, published in 2021, concluded that there was unlikely to be a single cause for the weekend effect. It pointed to the importance of case mix and concluded that the effect is unlikely to be an indicator of quality of hospital care.[4, 5]

In the perinatal field, studies have investigated pregnancy outcomes by day of the week, time of the year and time of day, particularly from the 1970s onwards. Analyses of perinatal mortality in England and Wales among births in the years 1970–76[6-8] and 1979–96[9] showed higher mortality rates among babies born at weekends, but could only estimate crude rates, meaning no conclusions could be drawn. Studies which were able to adjust for confounding produced inconsistent results. A study in Canada found slightly higher crude rates of stillbirths and neonatal deaths among births at weekends, but the difference disappeared after adjustment for gestational age,[10] and a study in Australia found no difference after adjustment for birthweight.[11] An analysis by day of the week in England published in 2015 concluded that perinatal mortality was highest at the weekend.[12] Some studies showed seasonal or short-term variation. Analyses of data for 1993–95 from Wales[13] and

For peer review only - http://bmjopen.bmj.com/site/about/guidelines.xhtml

Scotland[14], with relatively small numbers of deaths, suggested a possible association of mortality with the rotation of junior doctors to new posts in August. An analysis for England and Wales for 1979–96 showed seasonal variation, with perinatal mortality rates higher in winter than in summer.[9] Studies investigating time of day from Switzerland,[15, 16] Sweden[17, 18] and California[19] found higher mortality at night, although they reported varied risks in terms of type of perinatal death and time of night. An analysis of neonatal deaths at term in Scotland from 1985-2004 compared deaths among babies born from 9.00-17.00 on Mondays to Fridays to babies born outside these hours and days. It found higher rates of deaths ascribed to intrapartum anoxia among babies born 'out of hours' but no differences for other causes of death.[20]

The 2021 systematic review [4] recommended that further work should focus on underlying mechanisms and examine care processes in both hospital and the community. Such work has the potential to draw out the contributions of both case-mix and staffing questions to observed higher mortality. In this analysis, we have analysed neonatal mortality of babies born during working and non-working hours in English hospitals according to method of birth and onset of labour.

Methods

Data

This study uses linked data from birth registration, birth notification and maternity hospital episode statistics.

Information about births in England and Wales is recorded in several systems. Sociodemographic information is recorded at birth registration. Further information, including gestational age and time of birth, is recorded at birth notification when each baby is issued with a National Health Service (NHS) number. Information about care at birth in NHS hospitals in England is recorded in Maternity Hospital Episode Statistics (HES) within the mother-based HES deliveries file. Further information about the health of the baby and level of care required after birth is recorded in the baby-based HES baby file.

Following a series of pilot projects in collaboration with City, University of London, the Office for National Statistics (ONS) now routinely links birth registration and birth notification data. City, University of London has linked these data to HES and also to corresponding data for Wales to form the City Birth Cohort.[21] Authors had full access to the data from these previous efforts, and this study itself did not include further linkage.

Selection of data for analysis

This study uses a source dataset derived from the City Birth Cohort and consisting of all 6,054,536 singleton births occurring in NHS maternity units in England from 2005 to 2014 and with good links to HES. Derivation and analysis of linkage bias for this cohort has been described elsewhere.[22-24]

From this population, we removed births registered as stillborn and live births occurring before 22 weeks of gestational age. We used the ONS' modified Wigglesworth classification system[25] to classify neonatal deaths and removed deaths attributed to congenital anomalies. To derive the analysis population for modelling, we further removed births for which the time of birth was missing. We determined that recorded time of birth was subject to heaping on 5-minute intervals but that there were no other important accumulation points.

For peer review only - http://bmjopen.bmj.com/site/about/guidelines.xhtml

Statistical analysis

We used the mean and standard deviation to summarise continuous variables and *t*-test for comparisons between groups. We used percentages to summarise categorical variables and χ^2 test for comparisons between groups.

The primary outcome was cause-specific neonatal mortality attributed to asphyxia, anoxia or trauma using the ONS's modified Wigglesworth categories,[25, 26] as this category of death would be most likely to be affected by quality of care and staffing factors relevant to the 'weekend effect'; deaths attributed to intrapartum anoxia were responsible for elevated out-of-hours mortality in a study of over 1 million singleton live term births in Scotland between 1985 and 2004.[20] We also modelled all-cause neonatal mortality unattributed to congenital anomaly. Infant death was defined as death within a year of live birth and neonatal death was defined as death during the first 28 days after live birth. Postneonatal deaths, deaths after 28 days but under a year after live birth were not included as few of them are attributed to causes arising during or shortly after labour and birth (Table 1). They are also registered using a different death certificate from that used for stillbirths and neonatal deaths.[25]

We used an 8-day categorisation for day of birth which included Monday to Sunday and public holidays. Time of birth was categorised into daytime hours from 07:00 to 19:00 and night-time hours. A combination of day of birth and time of birth was used to classify births as occurring during 'working hours', defined as weekday daytime hours, and 'non-working hours', defined as all other times of the week and including all nights, weekends and holidays. In breaking down non-working hours further, the hours from midnight to 7am were classified according to the previous day, that is midnight to 7am on a Saturday was classified as a weeknight, and that period on a Monday was classified as a weekend/holiday night.

The dataset was stratified by method of birth and later analyses by mode of onset of labour. To determine the method of birth we used the Office of Population Census and Surveys (OPCS) procedure codes recorded in the standard HES record, supplemented by the HES 'maternity tail' variable DELMETH, as previously described.[22]

We used the HES maternity tail variable DELONSET to determine the mode of onset of labour. Models were constructed for each method of birth with onset of labour as a covariate.

We identified candidate covariates and confounders fitting univariable and multivariable logistic regression models and used a combination of forwards and backwards selection to determine inclusion of covariates in the models. Models were compared using the likelihood ratio test and the Bayesian information criterion (BIC).[27]

Long-term trends, and mother's age were characterised using natural cubic splines with the amount of smoothing chosen by minimising BIC as a function of degrees of freedom. Day of birth in the year was modelled using yearly and semestral harmonic terms. Binary contrasts referred to sex of the baby and changes between Greenwich Mean Time (GMT) and British Summer Time (BST). Birth attendants notifying births are instructed to notify gestational age in completed weeks from last menstrual period but some may have used gestational age as recorded by ultrasound as this has become routine. Birthweight was measured in grams and categorised into five levels for modelling. Ethnicity as recorded at birth notification was coded using 17 categories based on the ethnicity question in the 2001 Census of England and Wales. Parity was defined as nulliparous or multiparous by combining information from ONS and HES and by reviewing linked HES records' Mother IDs to determine whether there had been previous births.[28]

For peer review only - http://bmjopen.bmj.com/site/about/guidelines.xhtml

We accounted for geographic variation by adjusting for the former Strategic Health Authority (SHA) Region of England where the birth took place. Random effects terms were included in the models to allow for clustering of providers within the NHS trust where the birth took place. As the constituent hospital making up some trusts varied over the ten-year time period, maternity units were allocated to a single trust (the 'Assigned Trust') for the entire period, even if they were not part of this Trust for the entire period, as described in the project report.[28]

The risk of cause-specific and all-cause neonatal mortality was modelled fitting mixed-effects logistic regression models including a random effects term on the intercept to account for unobserved heterogeneity between Assigned Trusts.

During exploratory analyses we found associations between missing information for some variables and neonatal mortality. We also found non-random missingness patterns for key variables including gestational age and birthweight which was not suitable for imputation. For this reason, predictors were included in the models as categorical variables with a category for missing information. Data completeness, particularly for HES, has improved over time. We also observed this pattern in birthweight and gestational age data recorded at birth notification and registration; the percentage of births for which either birthweight or gestational age were missing was under 1%, well below the extent to which data were missing from HES. For around 15% births mode of onset of labour was unknown. They were included in the analyses that did not stratify by mode of onset, and included them in models as an unknown category rather than considering them as missing data.

Coding of all variables is shown in supplementary table 1. All analyses were performed in the ONS Secure Research Service with R version 3.6.1.

Public Involvement and Engagement

Public involvement and engagement (PI&E) have been at the core of the work undertaken to develop and analyse the City Birth Cohort using a documented 'three-tier' approach [25]. Two service user representatives were involved in the earlier NIHR-funded research from the outset as co-applicants. At the design stage, they contributed to the funding application and once funding was obtained they led the public engagement and involvement aspects of the project and helped to direct the focus of the research. Four service users were recruited to the study advisory group to oversee the direction of research and to provide a women-focused perspective. The groundwork of service user engagement for this analysis of birth outcomes according to time of day, and the potential impact of a 'weekend effect' was laid at the initial stages of the data linkage work. Four workshops were held regionally inviting maternity service user representatives to hear about the proposed work and to give their views. As a data analysis study, no recruitment to the study was required, and the participants were unseen, having contributed their data through routine data systems. This raised issues of data privacy and consent which were addressed at the workshops to ensure that the work involving patient-identifiable maternity records had a social licence from women whose data was recorded within the systems being analysed. To develop research questions and outcomes measures, women were asked about their experience of 'out of hours' care and their suggestions for further analyses and future research.

Subsequently, as the project developed, and in the ESRC-funded research described here, service user representatives from Maternity Voices Partnerships (MVPs) became involved. The PI&E strategy was based on the same three-tier model as developed earlier in the project [25] Our PI&E lead presented previous findings and our new plans at a national conference for MVP members in order

For peer review only - http://bmjopen.bmj.com/site/about/guidelines.xhtml

to collect the views of a wider constituency of parents, and also invited expressions of interest in joining a new PI&E advisory group. Three people (White British, Black British and Asian British) joined the group. Another MVP member joined our external advisory group at a later date. The PI&E group has met with the PI&E lead and data analyst regularly throughout the project to discuss progress and findings, including moving online during the COVID-19 pandemic. They have raised issues, such as the level of detail available on ethnicity, which have fed into the models, and stressed the importance of contextualising messages for both lay and clinical audiences. Group members have also worked with the PI&E lead and data analyst to develop guides to publicly available datasets and reports on five topics of particular concern to them: ethnicity in maternity statistics, miscarriage, birth interventions, prematurity and smoking in pregnancy. They also presented their learning, experiences and recommendations for future data intensive research at a dissemination event for parents and service user representatives. A second, multidisciplinary, event is planned in Spring 2023 and we will also share our results at future MVP conferences.

Figure 1: Population

[figure 1]

Results

Numbers and causes of deaths in the population

The source dataset consisted of 6,054,536 singleton births taking place in NHS maternity units in England between 1st January 2005 and 31st December 2014. We excluded 25,748 births recorded as stillborn (4.25 per 1000 births) and 1,782 births recorded as less than 22 weeks gestational age (0.03%) (Figure 1).

Numbers of neonatal and infant deaths subdivided by Wigglesworth cause of death categories are shown in Table 1. We further excluded 6,494 births recorded as infant deaths attributed to congenital anomalies. The population of live singleton births born after 22 weeks of gestational age and excluding deaths attributed to congenital anomalies had an overall infant mortality rate of 2.27 per 1000 live births and an overall neonatal mortality rate of 1.50 per 1000 live births (*N* = 6,020,512). There were 1,636 infant deaths attributed to asphyxia, anoxia or trauma, of which 1,494 were in the neonatal period. The neonatal mortality rate for deaths attributed to asphyxia, anoxia or trauma was 0.25 per 1000 live births.

Table 1. Neonatal and infant deaths by modified Wigglesworth cause of death categories

Causes of death	Neonatal deaths, *n*	Postneonatal deaths, *n*	Total infant deaths, *n*
**Cause arises before the onset of labour:**			
Congenital anomalies	4,070	2,424	6,494
Antepartum infections	489	48	537
Immaturity related conditions	6,178	864	7,042
**Cause arises during, or shortly after labour and birth:**			
Asphyxia, anoxia or trauma	1,494	142	1,636

For peer review only - http://bmjopen.bmj.com/site/about/guidelines.xhtml

**Cause arises after birth:**			
External conditions	44	276	320
Infections	237	864	1,101
Other specific conditions	111	200	311
Sudden infant deaths	190	1,048	1,238
**Unclassified:**			
Other conditions	239	1,191	1,430
Missing	25	35	60
**Total**	**13,077**	**7,092**	**20,169**
**Mortality rate per 1000 live births including congenital anomalies**	**2.17**	**1.18**	**3.35**
**Mortality rate per 1000 live births excluding congenital anomalies**	**1.50**	**0.78**	**2.27**

Characteristics of population for analysis of timing of birth

To derive the analysis population, we further excluded 55,864 births that were missing information about time of birth. We did not find any evidence of association between missing time of birth and neonatal mortality (Chi squared $p = 0.75$).

Baseline characteristics in the analysis population ($N = 5,964,648$) were stratified by whether births took place inside weekday working hours (weekdays 07:00 to 19:00) or outside working hours (nights, weekends and holidays) (Table 2). The proportion of births that took place outside of weekday working hours during nights, weekends or holidays was 61.0%, lower than the 65.1% of hours that were outside working hours. The standardised mean difference (SMD) was small for all characteristics (<0.10), except for mother's age, parity and previous caesareans. Among multiparous women, 32.0% of births during working hours were to people who had previously had a caesarean compared with 10.5% of those during non-working hours. This is likely to reflect elective repeat caesareans which have previously been shown to take place primarily on weekday mornings 9–11am.[22] Similarly, caesarean rates are higher among older women which could explain the higher proportions of births during working hours among older age groups.

Table 2. Characteristics of births in analysis population inside and outside of weekday working hours

Characteristic	Level	Working hours	%	Non-working hours	%	*p*-value	SMD
N		2,328,731		3,635,917			
Baby sex	F	1,134,931	48.7	1,766,765	48.6	0.001	0.003
	M	1,193,800	51.3	1,869,152	51.4		
Gestational age	22 to 28 weeks	6,764	0.3	12,025	0.3	<0.001	0.063
	28 to 32 weeks	15,219	0.7	21,832	0.6		
	32 to 37 weeks	115,228	4.9	175,908	4.8		
	37 to 42 weeks	2,097,955	90.1	3,236,055	89.0		
	42 weeks and over	76,852	3.3	163,639	4.5		
	Missing	16,713	0.7	26,458	0.7		
Birthweight	Under 2,500	129,603	5.6	204,111	5.6	<0.001	0.012

For peer review only - http://bmjopen.bmj.com/site/about/guidelines.xhtml

	2,500-2,999	369,786	15.9	589,999	16.2		
	3,000-3,499	848,644	36.4	1,318,645	36.3		
	3,500-3,999	694,708	29.8	1,086,195	29.9		
	4,000 and over	272,305	11.7	415,955	11.4		
	Missing or unfeasible	13,685	0.6	21,012	0.6		
Mother's age	Under 20	115,846	5.0	220,044	6.1	<0.001	0.116
	20-24	401,146	17.2	711,565	19.6		
	25-29	618,854	26.6	1,019,383	28.0		
	30-34	684,650	29.4	1,025,162	28.2		
	35-39	407,151	17.5	539,755	14.8		
	Over 40	101,084	4.3	120,008	3.3		
Parity	Nulliparous	892,405	38.3	1,594,567	43.9	<0.001	0.114
	Moderate: 1 to 4	1,367,620	58.7	1,935,522	53.2		
	High: 5 to 9	66,080	2.8	101,595	2.8		
	Very high: 10 to 14	2,409	0.1	3,905	0.1		
	Extreme/unfeasible	169	0.0	257	0.0		
	Missing	48	0.0	71	0.0		
Ethnicity	White British	1,491,685	64.1	2,319,066	63.8	<0.001	0.019
	White Irish	14,809	0.6	22,483	0.6		
	Any other White background	175,116	7.5	274,915	7.6		
	Mixed White & Black Caribbean	23,427	1.0	38,851	1.1		
	Mixed White & Black African	14,880	0.6	23,188	0.6		
	Mixed White & Asian	24,680	1.1	38,546	1.1		
	Any other Mixed background	41,207	1.8	64,505	1.8		
	Asian Indian	71,267	3.1	111,751	3.1		
	Asian Pakistani	94,864	4.1	155,558	4.3		
	Asian Bangladeshi	32,892	1.4	53,091	1.5		
	Any other Asian background	37,163	1.6	56,125	1.5		
	Black Caribbean	21,566	0.9	35,681	1.0		
	Black African	74,878	3.2	111,342	3.1		
	Any other Black background	17,003	0.7	26,458	0.7		
	Chinese	11,253	0.5	18,536	0.5		
	Any other ethnic group	48,855	2.1	74,154	2.0		
	Not Known	133,186	5.7	211,667	5.8		
Region	North East	113,142	4.9	173,066	4.8	<0.001	0.022
	North West	314,194	13.5	490,345	13.5		
	Yorkshire/Humber	228,995	9.8	373,778	10.3		
	East Midlands	173,054	7.4	273,713	7.5		
	West Midlands	254,354	10.9	405,784	11.2		
	East of England	231,783	10.0	358,103	9.8		

For peer review only - http://bmjopen.bmj.com/site/about/guidelines.xhtml

	London	461,053	19.8	704,290	19.4		
	South East Coast	186,082	8.0	282,432	7.8		
	South West	188,108	8.1	299,581	8.2		
	South Central	177,966	7.6	274,825	7.6		
Marital status at birth registration	Joint registration different address	215,147	9.2	366,533	10.1	<0.001	0.054
	Joint registration same address	681,512	29.3	1,111,692	30.6		
	Sole registration	134,867	5.8	227,363	6.3		
	Within marriage	1,297,198	55.7	1,930,320	53.1		
	Missing	7	0.0	9	0.0		
Mother's country of birth	United Kingdom	1,743,087	74.9	2,729,074	75.1	<0.001	0.016
	Other Europe	185,255	8.0	296,498	8.2		
	Africa	128,846	5.5	190,826	5.2		
	The Americas and the Caribbean	36,325	1.6	53,466	1.5		
	Middle East and Asia	221,835	9.5	346,122	9.5		
	Oceania and Antarctica	10,477	0.5	15,475	0.4		
	Missing	1,453	0.1	2,228	0.1		
Previous caesarean birth (multiparous women only)	No	976,973	68.0	1,826,456	89.5	<0.001	0.544
	Yes	459,188	32.0	214,645	10.5		

Crude neonatal mortality rates

Crude rates of cause-specific neonatal mortality attributed to asphyxia, anoxia or trauma were higher during non-working hours compared with working hours (0.27 compared with 0.21 per 1000 live births, rate ratio 1.26, 95% confidence interval 1.13 to 1.40). The crude mortality rate ratio was not significantly different from 1 for spontaneous, instrumental or emergency caesarean births. The elevated rate in the overall population was driven by a large rate ratio among births recorded as planned caesareans but occurring in non-working hours (Table 3), an estimate which is likely to be highly biased due to misclassification, as we discuss below. Due to this issue of misclassification, we did not include planned caesareans in the modelling analyses. Results were similar for all-cause mortality, except that the crude mortality rate ratio was significantly below 1 for emergency caesarean births (0.86, 0.80 to 0.93), meaning a lower crude all-cause neonatal mortality rate during non-working hours compared with working hours (supplementary table 2).

Table 3. Crude cause-specific neonatal mortality attributed to asphyxia, anoxia or trauma by time and day of birth, stratified by mode of birth

	Neonatal deaths	Total live births	Rate*	Rate ratio	95% CI
**Total population**					
Working hours	499	2,328,731	0.21		
Non-working hours	981	3,635,917	0.27	1.26	1.13 to 1.4
**Spontaneous birth**					

For peer review only - http://bmjopen.bmj.com/site/about/guidelines.xhtml

Working hours	115	1,202,710	0.10		
Non-working hours	264	2,558,357	0.10	1.08	0.87 to 1.35
**Instrumental birth**					
Working hours	60	256,452	0.23		
Non-working hours	107	485,425	0.22	0.94	0.69 to 1.3
**Emergency caesarean**					
Working hours	301	311,218	0.97		
Non-working hours	587	554,974	1.06	1.09	0.95 to 1.26
**Planned caesarean**					
Working hours	21	553,117	0.04		
Non-working hours	16	29,873	0.54	14.11	7.25 to 26.94

* Per 1000 live births

Adjusted odds of neonatal mortality by type of birth

We fitted multivariable mixed-effects logistic regression models to isolate the effect of working hours from potential confounding by other factors associated with neonatal mortality attributed to asphyxia, anoxia or trauma. Our final models were adjusted for baby's sex, gestational age, birthweight, mode of onset of labour, geographical region, NHS trust, baby's ethnicity, baby's date of birth, maternal age and maternal parity, as well as yearly harmonic terms for day of year of the birth. After adjustment, there was no significant difference in the odds of mortality during non-working compared with working hours for spontaneous or instrumental births. The odds of neonatal mortality attributed to asphyxia, anoxia or trauma for emergency caesareans was significantly higher during non-working compared with working hours (OR, 1.21; 95% CI, 1.05 to 1.39) (Table 4). For all-cause neonatal mortality, the odds ratios were not significantly different from 1 for any mode of birth (supplementary table 3).

Table 4. Unadjusted and adjusted odds ratios for neonatal mortality attributed to asphyxia, anoxia or trauma during non-working hours compared with working hours, by type of birth

	Non-working hours compared with working hours			
	Unadjusted OR	95% CI	Adjusted OR*	95% CI
**Neonatal mortality attributed to asphyxia, anoxia or trauma**				
Spontaneous birth	1.08	0.87 to 1.34	1.09	0.87 to 1.35
Instrumental birth	0.94	0.69 to 1.29	0.96	0.70 to 1.32
Emergency caesarean	1.09	0.95 to 1.26	1.21	1.05 to 1.39

* Adjusted for baby's sex, gestational age, birthweight, mode of onset of labour, geographical region, NHS trust, baby's ethnicity, baby's date of birth, maternal age and maternal parity, as well as yearly harmonic terms for day of year of the birth and a natural spline for day of the study period.

Onset of labour for emergency caesarean births

We further stratified emergency caesareans by mode of onset of labour. The largest group of emergency caesareans occurred after spontaneous onset, followed by induced onset, and less than one fifth are emergency caesareans before the start of labour. The rates of neonatal mortality were highest among the group with no labour, and that is also the only group for which the rate of neonatal mortality was significantly higher in non-working hours compared to working hours (Table 5).

For peer review only

For peer review only - http://bmjopen.bmj.com/site/about/guidelines.xhtml

Table 5. Crude cause-specific neonatal mortality attributed to asphyxia, anoxia or trauma by time and day of birth, among emergency caesareans births, stratified by mode of onset of labour

	Neonatal deaths	Total live births	Rate*	Rate ratio	95% CI
**Spontaneous onset**					
Working hours	103	120,791	0.85		
Non-working hours	204	226,528	0.90	1.056	0.84 to 1.34
**Induced onset**					
Working hours	29	75,513	0.38		
Non-working hours	71	162,353	0.44	1.139	0.75 to 1.78
**No labour**					
Working hours	86	60,202	1.43		
Non-working hours	169	71,700	2.36	1.65	1.28 to 2.15

* Per 1000 live births

The odds of neonatal mortality attributed to asphyxia, anoxia or trauma for emergency caesareans without labour were two thirds higher for births during non-working compared with working hours, unaffected by adjustment for characteristics of the mother, baby and birth (OR, 1.66; 95% CI, 1.28 to 2.15) (Table 6). No significant difference in neonatal mortality attributed to asphyxia, anoxia or trauma by working hours was seen for emergency caesareans with spontaneous or induced onset.

Table 6. Unadjusted and adjusted odds ratios for neonatal mortality attributed to asphyxia, anoxia or trauma outside of working hours compared with working hours for emergency caesareans

	Non-working hours compared with working hours			
	Unadjusted OR	95% CI	Adjusted OR*	95% CI
**Spontaneous onset**	1.06	0.83 to 1.34	1.06	0.83 to 1.34
**Induced onset**	1.14	0.74 to 1.75	1.14	0.74 to 1.76
**Caesarean onset (no labour)**	1.65	1.27 to 2.14	1.66	1.28 to 2.16

* Adjusted for baby's sex, gestational age, birthweight, geographical region, NHS trust, baby's ethnicity, baby's date of birth, maternal age and maternal parity, as well as yearly harmonic terms for day of year of the birth.

Babies born by emergency caesarean without labour are a group at inherently high risk, so to estimate the effect of out-of-hours care we further adjusted the model for this group of births for obstetric risk factors. We compared obstetric risk factor characteristics during working and non-working hours (supplementary table 4) and included in the model those that improved fit. After adjustment, there remained 48% higher odds of neonatal mortality attributed to asphyxia, anoxia or trauma during non-working compared with working hours (OR 1.48; 95% CI 1.14 to 1.92) (Table 7). The full coefficients in the fully adjusted model are presented in supplementary table 5.

Table 7. Odds ratios for neonatal mortality attributed to asphyxia, anoxia or trauma during non-working hours compared with working hours for emergency caesareans without labour with additional adjustment for obstetric risk factors

	Non-working compared with working hours			
	OR adjusted for characteristics of mother, baby and birth*	95% CI	OR further adjusted for obstetric risk factors**	95% CI

For peer review only

**Emergency caesarean without labour**	1.66	1.27 to 2.14	1.48	1.14 to 1.92

* Adjusted for baby's sex, gestational age, birthweight, geographical region, NHS trust, baby's ethnicity, baby's date of birth, maternal age and maternal parity, as well as yearly harmonic terms for day of year of the birth. ** Further adjusted for placental abruption, maternal care for abnormality of pelvic organs, malpresentation of the fetus, preeclampsia, postpartum haemorrhage, and antepartum haemorrhage.

Timing within non-working hours

To understand better the circumstances in which births were at higher risk of neonatal mortality we disaggregated the "non-working hours" category to allow us to distinguish evenings from weekends and holidays. Among emergency caesarean births without labour, being born during daytime hours of 07:00 to 19:00 at the weekend or on a holiday held no greater risk of mortality attributed to anoxia, asphyxia or trauma than being born during daytime hours on a working weekday. By contrast, emergency caesarean birth without labour in the night-time held an increased risk both during weeknights (odds ratio = 1.56, 95%CI 1.15 to 2.11) and night-time at weekends and holidays (odds ratio = 1.75, 95%CI 1.24 to 2.47) (Table 8). There still was no association between mortality risk and working hours for spontaneous or instrumental birth or emergency caesareans with spontaneous/induced onset using the four-category timing variable (data not shown).

Table 8. Crude numbers and rates, and modelled odds ratios, for neonatal mortality attributed to asphyxia, anoxia or trauma during non-working hours compared with s weekday daytime working hours for emergency caesareans without labour

	**Neonatal deaths**	**Total live births**	**Crude rate***	**Adjusted** odds ratio**	**95% CI**
**Weekday daytime**	86	60202	1.43	*Ref*	*Ref*
**Weekday night-time**	86	33558	2.56	1.56	1.15 to 2.11
**Weekend/holiday daytime**	29	20068	1.45	0.95	0.63 to 1.46
**Weekend/holiday night-time**	54	18074	2.99	1.75	1.24 to 2.47

* Per 1000 live births

** Adjusted for baby's sex, gestational age, birthweight, geographical region, NHS trust, baby's ethnicity, baby's date of birth, maternal age and maternal parity, as well as yearly harmonic terms for day of year of the birth, and presence of obstetric risk factors (placental abruption, maternal care for abnormality of pelvic organs, malpresentation of the fetus, preeclampsia, postpartum haemorrhage, and antepartum haemorrhage).

We further investigated whether risk of death in non-working hours was significantly associated with gestational age, or year of birth. We found that the only gestational age category where non-working hours had a significant crude rate ratio for death attributed to anoxia, asphyxia or trauma compared to working hours was among term births (37 to 42 weeks). We found no evidence that the association of non-working hours with mortality differed by epoch of birth (characterised in two-year epochs and in five-year epochs across the period 2005 to 2014).

For peer review only

For peer review only - http://bmjopen.bmj.com/site/about/guidelines.xhtml

Absolute risks from emergency caesarean without labour during non-working hours

Following a suggestion from our PPI group, we calculated how many emergency caesareans without labour would have to occur out of hours to cause a neonatal death attributed to asphyxia, anoxia or trauma beyond those expected if all emergency caesareans without labour happened during working hours. This 'number needed to harm' (NNH) was calculated as

$$NNH = ((PEER \times (OR - 1)) + 1) \div (PEER \times (OR - 1) \times (1 - PEER))$$

where the patient expected event rate (PEER) was taken as the rate of neonatal death attributed to asphyxia, anoxia or trauma among emergency caesareans without labour during weekday working hours, 1.43 per 1000 live births (Table 5), and the odds ratios from the model further adjusted for obstetric risk factors were 1.56 for weekday night-time and 1.75 for weekend/holiday night-time (Table 8). The resulting NNHs were 1258 for weekday nights and 933 for weekend/holiday nights. This means that for every 1258 births by emergency caesarean without labour born on a weekday night, there would be one additional neonatal death attributed to asphyxia, anoxia or trauma above what would have happened if all those births had been in weekday working hours; for such births on a weekend/holiday night, there would be one additional neonatal death attributed to asphyxia, anoxia or trauma in every 933 births. This amounts to 46 additional neonatal deaths in England across the 10-year study period, or between 3 and 6 deaths per year (supplementary table 6). It constitutes 18% of the 255 neonatal deaths attributed to asphyxia, anoxia or trauma occurring among emergency caesareans without labour, 3% of the 1,494 neonatal deaths attributed to asphyxia, anoxia or trauma and 0.4% of all neonatal deaths.

Discussion

Statement of principal findings

Overall, this study did not find evidence of a higher risk of all-cause or cause-specific neonatal mortality for spontaneous or instrumental births, or for births by emergency caesarean after spontaneous or induced onset, when born outside of working hours.

Among babies coded as born by emergency caesarean without labour, which accounted for 2% of births in our analysis population, those born at night-time during the week had 56% higher odds of neonatal death attributed to asphyxia, anoxia or trauma when compared with those born during weekday working hours (defined as between 7am and 7pm Monday to Friday); those born at night-time during the weekend or on a holiday had 75% higher odds compared with those born in weekday working hours. In our dataset, with 71,700 births coded as emergency caesareans without labour outside working hours, we estimate that 46 excess deaths are likely to be attributable to being outside of working hours across the ten years.

For births recorded as planned caesareans, we found evidence of an inflated rate ratio for all-cause and cause-specific mortality outside of working hours which may be related to data recording and requires further consideration. Only 29,873 of the 582,990 planned caesareans in our analysis population (5%) took place outside of working hours. Among 'planned caesareans', the underlying population differed according to working hours: planned caesareans usually occur in working hours, and those 'planned caesareans' that occur in non-working hours are likely to involve complications. An audit of caesarean section in Scotland in 1994/95 found that elective caesareans outside the hours of 9am to 6pm occurred mainly where women were booked for an elective caesarean but went into labour before the planned date.[29] A comparison of OPCS procedure codes and the

For peer review only - http://bmjopen.bmj.com/site/about/guidelines.xhtml

DELMETH variable from the HES maternity tail published in 2013 found that the largest group of inconsistencies was those where one source recorded an emergency caesarean and the other recorded a planned caesarean.[26]

Strengths and weaknesses of the study &

Strengths and weaknesses in relation to other studies, discussing important differences in results

This study used a large, linked dataset which brings together information on birth registration, birth notification and hospital maternity data for over 6 million births over ten years. The large cohort allowed for stratification by distinct care pathways based on both onset of labour and mode of birth, and for the opportunity to examine mortality attributed to asphyxia, anoxia or trauma, most likely to be affected by care at birth but comprising approximately 11% of neonatal deaths. The study in Scotland found a raised risk of mortality attributed to anoxia in term births [20] but because Scotland has a much smaller population than England , the data related to 1,039,560 live births over the 20 years 1985 to 2004 and the numbers were not large enough to stratify by onset of labour and type of birth. That study used data from the Scottish Stillbirth and Infant Death Survey in which information from death certificates was supplemented by further information recorded in clinical settings and classified using a modification of the Wigglesworth classification. The definition of anoxia was 'broad, including hypoxia, acidosis and asphyxia'.[20] The ONS modified Wigglesworth classification[25] had been designed to classify conditions recorded on stillbirth and neonatal death certificates and does not use data from other sources. It groups together anoxia, asphyxia and trauma and so the inclusion criteria do not differ significantly from those used in the earlier study.

The analysis of Scottish data also included only term births. Our data also allowed adjustment for obstetric risk factors. Our findings confirm those of this previous report but further identify a specific cohort of births at risk: the subset of births born by emergency caesarean without labour. Overall, mortality for babies born in NHS hospital units is largely consistent by time and day.

The nations of the UK are unusual in stratifying caesarean sections into elective/planned and emergency, while in many other countries the subdivision is into whether they take place before or in labour.[30] The subgroup we have identified would not have been visible if the latter categorisation had been used. The audit of caesarean section in Scotland in 1994/95 found, at a time when the overall caesarean section rate was lower, that 14.1% of caesarean sections were classified as emergency sections before the onset of labour and that these were mainly attributed to fetal growth restriction or fetal distress.[29]

Given the large number of variables available in the linked datasets, we were able to adjust for relevant covariates including obstetric risk factors. We have also adjusted for the seasonal and temporal nature of births and neonatal mortality over the study period, reported in our previous analyses of this dataset.[22]

A study using data from Maternity HES to analyse perinatal mortality by day of the week concluded that mortality was higher at weekends.[31] Without linkage to birth notification, it was unable to take account of the time of day and the authors' analysis and interpretation of their results was highly criticised in subsequent rapid responses.[32-34] Like past analyses of rates of stillbirth and

For peer review only - http://bmjopen.bmj.com/site/about/guidelines.xhtml

For peer review only

perinatal mortality in England and Wales, this analysis made no distinction between intrapartum and antepartum stillbirths, despite the fact that the majority of stillbirths are antepartum and are therefore most are unlikely to be affected by care at birth.

In contrast to other studies, we removed stillbirths from our analyses. We had planned to investigate intrapartum stillbirths but the problem here is to identify the stillbirths which were definitely intrapartum in the presence of substantial numbers of stillbirths for which it is unclear whether they occurred before or during labour. One of our advisors, the late Martin Ward Platt, suggested that we obtain confidential enquiry data to supplement the information from stillbirth registration certificates, following the precedent of the Scottish Stillbirth and Infant Death Survey, but it proved impossible to obtain a consistent series of data because of the many changes which took place in the confidential enquiries over the years 2005 to 2014. Permission was obtained from the Healthcare Quality Improvement Partnership to access data compiled by CEMACH and CMACE for births in the years 2005 to 2010, in which there were a number of year-to-year changes.[21] More substantial changes were made from 2012 when responsibility for the confidential enquiry programme passed to Mothers and Babies: Reducing Risk through Audits and Confidential Enquiries across the UK (MBRRACE-UK). Its most recent report found that of the 2040 stillbirths in England in 2019, 88 per cent were antepartum. Of the remainder 160 were classified as occurring intrapartum and 76 being of unknown timing.[35] In view of this continuing difficulty, we did not continue our plans to analyse Intrapartum stillbirths. Studies have used 9am to 5pm to represent working hours, but it has been suggested that this may not reflect working hours in the NHS. We used a wider definition of working hours 7am to 7pm to capture this and to reduce the potential for misclassification.

A key strength is our patient engagement – we have presented the number needed to harm through recommendations from maternity users who felt that odds ratio and relative risks were not accessible for all to understand and did not fully capture rarity of events and absolute risk. This is also important for planning and strategies. To our knowledge, this is the first time that the NNH has been estimated for infant mortality in relation to hospital working hours and birth type.

As with all observational studies, there is a potential for unmeasured confounding despite adjustment for multiple covariates. There is also potential for variation in reporting which can vary between trusts and over time, although the quality and completeness of HES improved over the period covered by this study. Future analyses are needed to confirm if these results persisted after the study period as the introduction in 2015 of a second national system, the Maternity Services Dataset may have competed for staff time available for data recording. Investigations of the 'weekend effect' assume that timing of birth is associated with availability of specialist care, but we did not have any data to directly measure that availability. The size and completeness of our dataset gives us confidence in the accuracy of our findings for NHS births in England as a whole, although variation within England will not be captured in this estimate.

We excluded planned caesareans from our modelling analyses as there was an important conceptual difference or these births regarding birth during/out of working hours. We suspect that the crude findings related to planned caesareans was due to misclassification of actual emergency caesareans as 'planned', but there is little way to verify this without booking information and this requires further investigation by other means. There could be an important subgroup of women who are identified as at risk at booking but then progress to become emergencies. We were unable to assess using the administrative data in this study whether this is related to any differences in care in and out of hours.

For peer review only - http://bmjopen.bmj.com/site/about/guidelines.xhtml

Meaning of the study: possible explanations and implications for clinicians and policymakers

Overall, out of hours care is not associated with a raised risk of neonatal mortality. Policy should focus on making arrangements for the small subset of emergencies where there may be an association rather than regarding all out of hours births as dangerous. Such arrangements may regard antenatal monitoring of or advice on healthcare-seeking behaviour for parents of particularly vulnerable babies.

Our NNH finds that over a ten-year period there were approximately 49 excess neonatal deaths attributed to anoxia, asphyxia or trauma among babies born out of hours by emergency caesarean without labour.

Emergency caesareans without labour make up approximately 2% of all births over the study period. Compared with other care pathways, they have by far the highest crude rates of neonatal mortality both overall and of mortality attributed to anoxia, asphyxia or trauma, both in and out of working hours.

As such, these births represent a cohort of mothers and babies with high risk, emergent conditions that are, by nature, likely to be unpredictable. The 2017 MBRRACE report on term, singleton, intrapartum stillbirth and intrapartum-related neonatal deaths reported that many of the deaths they reviewed followed pregnancies classified as low-risk.[36] That report also found that in at least a quarter of the 78 cases reviewed, sampled randomly from all eligible deaths, staffing capacity issues played a role in the death. A systematic review of articles on weekend mortality did not establish clear reasons for the higher weekend mortality for general NHS A&E care.[4] That review found no weekend effect in maternity admissions [37] although these were not disaggregated as we have done, and the weekday/weekend difference may elicit different findings that the working/non-working hours classification that we used. That study also did not include comparisons of daytime and night-time, which our results suggest may be more important than the weekday/weekend distinction for the small group of births where working hours is associated with higher risk. The present study presents a comparable pattern of increased risk for births that reflect 'A&E-like' emergency maternal care, women not in labour but with conditions which indicate an immediate caesarean. Possible factors of influence might include differences in healthcare-seeking behaviour out of hours, availability of staff out of hours to take calls querying symptoms and transport to maternity units being more difficult out of hours. This could lead to differences in case presentation with maternity users presenting with conditions that require emergency caesareans later than during working hours.

Unanswered questions and future research

We have found that women facing an emergency requiring caesarean birth without labour have a small increased risk of neonatal mortality when giving birth at night. However, the reasons behind this remain unclear.

Future research should focus on characterising the cohort of women who have an emergency caesarean without labour, whether there are aspects of their care pathways or care-seeking behaviour that would be amenable to intervention, and whether a planned caesarean would have been an option.

For peer review only - http://bmjopen.bmj.com/site/about/guidelines.xhtml

Analyses by NHS trust would not include sufficient numbers of deaths to look at mortality outcomes but could provide useful information on rates of emergency caesareans in and out of hours and ideally could link to information on staff-to-patient ratios. We had hoped to do this in our earlier project using NHS workforce statistics, but found that they were too inconsistent.[21] Healthcare-seeking behaviour should be investigated and whether widening access to out of hours 'helplines' could be useful.

License for publication

The Corresponding Author has the right to grant on behalf of all authors and does grant on behalf of all authors, a worldwide licence to the Publishers and its licensees in perpetuity, in all forms, formats and media (whether known now or created in the future), to i) publish, reproduce, distribute, display and store the Contribution, ii) translate the Contribution into other languages, create adaptations, reprints, include within collections and create summaries, extracts and/or, abstracts of the Contribution, iii) create any other derivative work(s) based on the Contribution, iv) to exploit all subsidiary rights in the Contribution, v) the inclusion of electronic links from the Contribution to third party material where-ever it may be located; and, vi) licence any third party to do any or all of the above.

The Submitting Author has the right to grant and does grant on behalf of all authors of the Work an exclusive licence on a worldwide, perpetual, irrevocable, royalty-free basis to BMJ Publishing Group Ltd ("BMJ") and its licensees.

The Submitting Author accepts and understands that any supply made under these terms is made by BMJ to the Submitting Author unless you are acting as an employee on behalf of your employer or a postgraduate student of an affiliated institution which is paying any applicable article publishing charge ("APC") for Open Access articles. Where the Submitting Author wishes to make the Work available on an Open Access basis (and intends to pay the relevant APC), the terms of reuse of such Open Access shall be governed by a Creative Commons licence – details of these licences and which licence will apply to this Work are set out in our licence referred to above.

Ethics approval

Ethics approval 05/Q0603/108 and subsequent substantial amendments were granted by East London and City Local Research Ethics Committee 1 and its successors.

Use of patient identifiable data without consent

Permission to use patient identifiable data without consent under Section 60 of the Health and Social Care Act 2001 was initially granted by the Patient Information Advisory Group PIAG 2-10(g)/2005. Renewals and amendments under Section 251 of the Health Service (Control of Patient Information) Regulations 2002 were granted by its successor bodies, the Ethics and Confidentiality Committee of the National Information Governance Board and the Confidentiality Advisory Group of the Health Research Authority.

A second permission CAG 9-08(b)2014 to use patient identifiable data without consent under Section 251 of the Health Service (Control of Patient Information) Regulations 2002 to create a research database held at the Office for National Statistics for analyses relating to inequalities in the outcome of pregnancy and to inform maternity service users about the outcome of midwifery,

For peer review only - http://bmjopen.bmj.com/site/about/guidelines.xhtml

obstetric and neonatal care was granted by the Confidentiality Advisory Group of the Health Research Authority.

Use of the Office for National Statistics Secure Research Service

Permission to access data from the Office for National Statistics in the VML, now known as the Secure Research Service was granted by ONS's Microdata Release Panel, now superseded by its Research Advisory Panel. All members of the research team successfully applied for ONS Approved Researcher Status. The work is covered by the following disclaimer:

'This work was produced using statistical data from ONS. The use of the ONS statistical data in this work does not imply the endorsement of the ONS in relation to the interpretation or analysis of the statistical data. This work uses research datasets which may not exactly reproduce National Statistics aggregates.'

Permission to use data from the Health and Social Care Information Centre.

Permission to link and analyse data held by the Health and Social Care Information Centre, now known as NHS Digital, was granted under Data Sharing Agreements NIC-273840-N0N0N and subsequently under DARS-NIC-10094-P6P4B-v6.7 – Linkage, analysis and dissemination of national birth and maternity data for England and Wales

Transparency statement

The manuscript's guarantor affirms that this manuscript is an honest, accurate, and transparent account of the study being reported; that no important aspects of the study have been omitted; and that any discrepancies from the study as planned have been explained.

Funding

The work described here was funded by the ESRC under its Secondary Data Analysis Initiative. Implications of time of day and day of the week for the outcome of birth' (ES/S010785/1) It draws on earlier work funded by NIHR 'Births and their outcomes by time, day and year: a retrospective birth cohort data linkage study' HSDR 12/136/93. The views expressed are those of the authors and not necessarily those of the ESRC.

Contributor and guarantor information

AM, RP and MCB conceived and designed the analysis and AM is the guarantor of the overall content.

RP led the patient and public involvement activities.

LC, CG and MCB conducted the analysis.

All authors drafted or gave substantive comments on the manuscript. The corresponding author attests that all listed authors meet authorship criteria and that no others meeting the criteria have been omitted. The authors are grateful to Miranda Scanlon and Dharmintra Pasupathy for comments on the manuscript and to the members of the PI&E group and our project advisory group for their guidance and input.

For peer review only

Competing interests

All authors have completed the ICMJE uniform disclosure form at http://www.icmje.org/disclosure-of-interest/ and declare: no support for the submitted work from any organisation other than their employers and research grant-making bodies; no financial relationships with any organisations that might have an interest in the submitted work in the previous three years; and no other relationships or activities that could appear to have influenced the submitted work.

Data Sharing Statement

The authors do not have permission to supply data or identifiable information to third parties, including other researchers, but they have permission under Regulation 5 of the Health Service (Control of Patient Information) Regulations 2002 to analyse confidential patient information for England and Wales without consent and create a research database which could be accessed by other researchers using the Secure Research Service at the Office for National Statistics. The information held includes the subset of data and the R computing code used to undertake the analyses described in this article. Anyone wishing to access the linked datasets for research purposes should apply to the Office for National Statistics and NHS Digital as well as to the Health Research Authority via the Confidentiality Advisory Group to access confidential patient information without consent. In the first instance, enquiries about access to the data should be addressed to the principal investigator, Alison Macfarlane (A.J.Macfarlane@city.ac.uk).

References

1. Bray, B.D. and A. Steventon, *What have we learnt after 15 years of research into the 'weekend effect'?* BMJ Quality & Safety, 2016.
2. Wise, J., *The weekend effect—how strong is the evidence?* BMJ, 2016. **353**.
3. Department of Health, *The Government's mandate to NHS England for 2016-17*. 2015: London.
4. Bion, J., et al., *Increasing specialist intensity at weekends to improve outcomes for patients undergoing emergency hospital admission: the HiSLAC two-phase mixed-methods study*, in *Health Services and Delivery Research*. 2021, NIHR Journals Library: Southampton (UK).
5. Chen, Y.F., et al., *The magnitude and mechanisms of the weekend effect in hospital admissions: A protocol for a mixed methods review incorporating a systematic review and framework synthesis.* Syst Rev, 2016. **5**.
6. Macfarlane, A., *Variations in number of births and perinatal mortality by day of week in England and Wales.* BMJ, 1978. **2**(6153): p. 1670-3.
7. Macfarlane, A., *Day of the week variations in numbers of births and perinatal mortality rates.* Journal of Maternal and Child Health, 1979. **4**: p. 415-416.

For peer review only - http://bmjopen.bmj.com/site/about/guidelines.xhtml

8. Macfarlane, A. and P. Thew, *Births: the weekly cycle.* Population Trends, 1978. **13**: p. 23-24.

9. Hawe, E., A. Macfarlane, and J. Bithell, *Daily and seasonal variation in live births, stillbirths and infant mortality in England and Wales, 1979-96.* Health Statistics Quarterly, 2001. **09**: p. 5-15.

10. Luo, Z.-C., et al., *Risks of stillbirth and early neonatal death by day of week.* Canadian Medical Association Journal, 2004. **170**(3): p. 337-341.

11. Mathers, C.D., *Births and perinatal deaths in Australia: variations by day of week.* J Epidemiol Community Health, 1983. **37**(1): p. 57-62.

12. Palmer, W.L., A. Bottle, and P. Aylin, *Association between day of delivery and obstetric outcomes: observational study.* BMJ, 2015. **351**: p. h5774.

13. Stewart, J.H., J. Andrews, and P.H. Cartlidge, *Numbers of deaths related to intrapartum asphyxia and timing of birth in all Wales perinatal survey, 1993-5.* BMJ, 1998. **316**(7132): p. 657-660.

14. Chalmers, J.W.T., et al., *Scottish data on intrapartum related deaths are in same direction as Welsh data.* BMJ, 1998. **317**(7157): p. 539.

15. Paccaud, F., B. Martin-Beran, and F. Gutzwiller, *Hour of birth as a prognostic factor for perinatal death.* Lancet, 1988. **331**(8581): p. 340-3.

16. Ruffieux, C., A. Marazzi, and F. Paccaud, *The circadian rhythm of the perinatal mortality rate in Switzerland.* American journal of epidemiology, 1992. **135**(8): p. 936-952.

17. Luo, Z.C. and J. Karlberg, *Timing of birth and infant and early neonatal mortality in Sweden 1973-95: longitudinal birth register study.* BMJ, 2001. **323**(7325): p. 1327-30.

18. Stephansson, O., et al., *Time of birth and risk of intrapartum and early neonatal death.* Epidemiology, 2003. **14**(2): p. 218-22.

19. Gould, J.B., C. Qin, and G. Chavez, *Time of birth and the risk of neonatal death.* Obstet Gynecol, 2005. **106**(2): p. 352-8.

20. Pasupathy, D., et al., *Time of birth and risk of neonatal death at term: retrospective cohort study.* BMJ, 2010. **341**: p. c3498.

21. Macfarlane, A., et al., *Births and their outcomes by time, day and year: a retrospective birth cohort data linkage study.* Health Services and Delivery Research, 2019. **7**(18).

For peer review only

For peer review only - http://bmjopen.bmj.com/site/about/guidelines.xhtml

22. Martin, P., et al., *Timing of singleton births by onset of labour and mode of birth in NHS maternity units in England, 2005–2014: A study of linked birth registration, birth notification, and hospital episode data.* PLOS ONE, 2018. **13**(6): p. e0198183.

23. Dattani, N. and A. Macfarlane, *Linkage of Maternity Hospital Episode Statistics data to birth registration and NHS Numbers for births in England 2005–2014: methods. A population-based birth cohort study.* BMJ Open, 2018. **8**: p. e017897.

24. Harper, G., *Linkage of Maternity Hospital Episode Statistics data to birth registration and notification records for births in England 2005–2014: quality assurance of linkage of routine data for singleton and multiple births.* BMJ Open, 2018. **8**: p. e017898.

25. Dattani, N. and S. Rowan, *Causes of neonatal deaths and stillbirths: a new hierarchical classification in ICD-10.* Health Statistics Quarterly, 2002. **15**.

26. Wigglesworth, J.S., *Monitoring perinatal mortality. A pathophysiological approach.* Lancet, 1980. **2**(8196): p. 684-6.

27. Kuha, J., *AIC and BIC: Comparisons of Assumptions and Performance.* Sociological Methods & Research, 2004. **33**(2): p. 188-229.

28. National Institute for Health Research. *Births and their outcome: analysing the daily, weekly and yearly cyles and their implications for the NHS: Study Documentation*. 2017 15 November 2017]; Available from: https://www.journalslibrary.nihr.ac.uk/programmes/hsdr/1213693/#/.

29. McIlwaine, G., et al., *Caesarean Section in Scotland 1994/5: a National Audit.* 1996.

30. Euro-Peristat project with SCPE and Eurocat, *Euro-Peristat Project: European Perinatal Health Report. Core indicators of the health and care of pregnant women and babies in Europe in 2015*. 2018.

31. Aylin, P., et al., *Weekend mortality for emergency admissions. A large, multicentre study.* Quality and Safety in Health Care, 2010. **19**(3): p. 213-217.

32. Freudenthal, B. *Editorial double-standards for Dr Foster - a major fail for BMJ. BMJ Rapid Response 25 November 2015*. 2015; Available from: http://www.bmj.com/content/351/bmj.h5774/rr-8.

For peer review only - http://bmjopen.bmj.com/site/about/guidelines.xhtml

33. Macfarlane, A., et al. *Re: Association between day of delivery and obstetric outcomes: observational study, BMJ Rapid Response 25 November 2015*. 2015; Available from: http://www.bmj.com/content/351/bmj.h5774/rr-7.

34. Richmond, D., et al. *Re: Assocation between day of delivery and obstetric outcomes: observational study. BMJ Rapid Response 25 November 2015*. 2015; Available from: http://www.bmj.com/content/351/bmj.h5774/rr-13.

35. Draper, E., et al., *MBRRACE-UK Perinatal Mortality Surveillance Report, UK Perinatal Deaths for Births from January to December 2019*. 2021, The Infant Mortality and Morbidity Studies, Department of Health Sciences, University of Leicester: Leicester.

36. Draper, E., J. Kurinczuk, and S. Kenyon (eds), *MBBRACE-UK Perinatal Confidential Enquiry: Term, singleton, intrapartum stillbirth and intrapartum-related neonatal death*, MBBRACE-UK, Editor. 2017.

37. Chen, Y.-F., et al., *Magnitude and modifiers of the weekend effect in hospital admissions: a systematic review and meta-analysis.* BMJ Open, 2019. **9**(6): p. e025764.

For peer review only

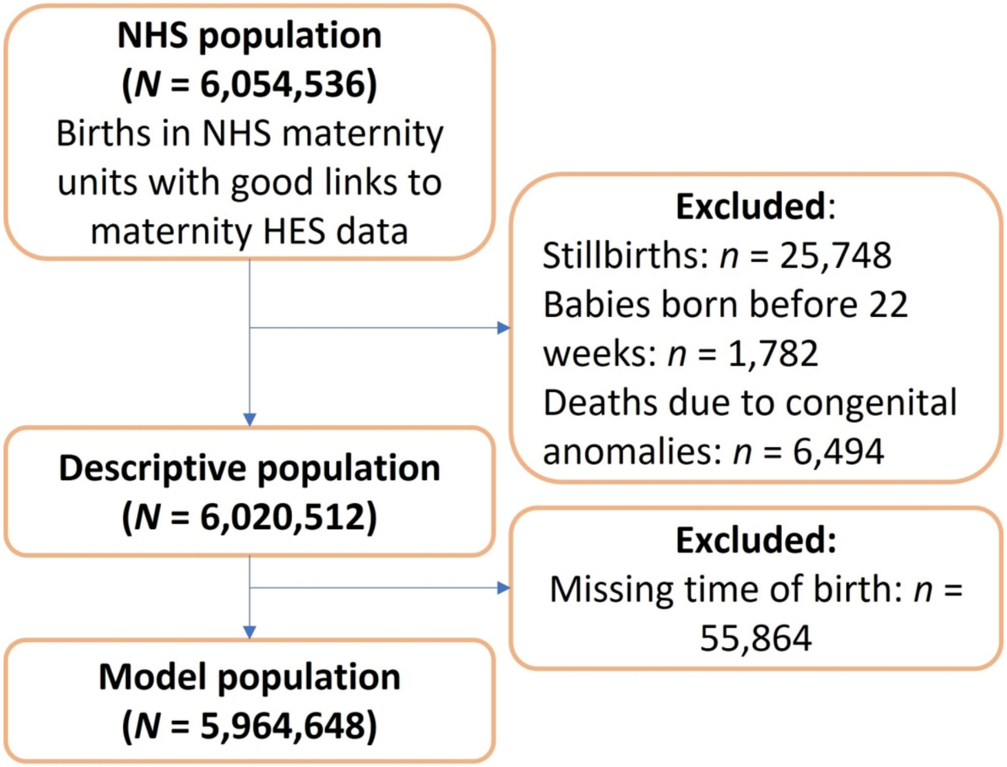

Population

155x120mm (300 x 300 DPI)

Neonatal mortality in NHS maternity units by timing and method of birth: a retrospective linked cohort study

Lucy Carty, Christopher Grollman, Rachel Plachcinski, Mario Cortina-Borja and Alison Macfarlane

[Citation detail]

DOI: [Article DOI]

Supplementary material

Coding of all variables used in the analysis

Coding of variables used in the analysis.

Table 1. Coding of all variables used in the analysis

Variable in analysis	Original format	Coding
Baby's date of birth	Calendar date	Continuous from first to last day of study
Day in year of baby's date of birth	Calendar date	Continuous from 1 to 365 or 366
Clock go forward/back	Calendar date	Binary for whether clocks went forward/back that day
Working hours (binary)	Time of birth, calendar date	Binary:<br>Working hours = 0700 to 1859 inclusive, Monday to Friday except public holidays<br>Non-working hours = all other times
Working hours (4 categories)	Time of birth, calendar date	Four categories:<br>Working hours (weekday daytime) = 0700 to 1859, Monday to Friday except public holidays<br>Weekday night-time = 1900 to 2359, Monday to Friday except public holidays; 0000 to 0659 Tuesday to Friday except public holidays<br>Weekend/holiday daytime = 0700 to 1859 Saturday, Sunday or public holiday<br>Weekend/holiday night-time = 1900 to 2359, Saturday, Sunday or public holiday; 0000 to 0659 Saturday, Sunday, public holiday, Monday or day after public holiday
Gestational age	Completed weeks since last menstrual period, or by ultrasound	Grouped in categories:<br>22 to 28 weeks; 28 to 32 weeks; 32 to 37 weeks; 37 to 42 weeks; 42 weeks and over; Missing

For peer review only - http://bmjopen.bmj.com/site/about/guidelines.xhtml

Birthweight	Birthweight in grammes	Grouped in categories: <br><2500; 2500–2999; 3000–3499; 3500–3999; 4000+
Age of mother	Fractional years	Continuous in completed years
Parity	Number of previous births	Binary nulliparous/multiparous
Ethnicity	17 categories based on the ethnicity question in the 2001 Census of England and Wales	Seventeen categories: <br>White British; White Irish; Any other White background; Mixed White & Black Caribbean; Mixed White & Black African; Mixed White & Asian; Any other Mixed background; Asian Indian; Asian Pakistani; Asian Bangladeshi; Any other Asian background; Black Caribbean; Black African; Any other Black background; Chinese; Any other ethnic group; Not Known
Region	Ten Strategic Health Authority regions in the NHS for the majority of the study period	Ten categories: North East; North West; Yorkshire/Humber; East Midlands; West Midlands; East of England; London; South East Coast; South West; South Central
Marital status at birth registration	Categories recorded at birth registration	Four categories: <br>Joint registration with different address; Joint same address; Sole registration; Within marriage
Mother's place of birth	Countries recorded at birth registration	Grouped into seven categories: <br>United Kingdom; Other Europe; Africa; The Americas and the Caribbean; Middle East and Asia; Oceania and Antarctica; Missing
Previous caesarean birth	Whether present (yes/no)	Binary no/any previous caesarean birth
Obstetric complications*	Whether present (yes/no)	Binary not present/present

* Hypertension, Preeclampsia, Eclampsia, Diabetes, Gestational diabetes, Malpresentation of foetus, Maternal care for disproportion, Maternal care for abnormality of pelvic organs, Polyhydramnios, Oligohydramnios, Premature rupture of membranes, Placental disorders, Placenta praevia, Premature separation of placenta, Antepartum haemorrhage, Obstructed labour, Intrapartum haemorrhage, Umbilical cord complications

Crude all-cause neonatal mortality rates

Crude all-cause neonatal mortality rates in the analysis population were higher during non-working hours compared with working hours (1.58 vs 1.36 per 1000 live births, rate ratio 1.17, 95% confidence interval 1.12 to 1.22) (Table 2). The crude mortality rate ratio was not significantly different from 1 for spontaneous or instrumental births and was below 1 for emergency caesarean births (0.86, 0.80 to 0.93), meaning lower crude all-cause neonatal mortality rate during non-working hours compared with working hours (3.13 vs 3.62 per 1000 live births).

For peer review only - http://bmjopen.bmj.com/site/about/guidelines.xhtml

Table 2. Crude all-cause neonatal mortality by time and day of birth, stratified by method of birth

	Living at 28 days	Neonatal death	Total	Rate*	Rate ratio	95% CI
**Total population**						
Working hours	2325569	3162	2328731	1.36		
Non-working hours	3630159	5758	3635917	1.58	1.17	1.12 to 1.22
**Spontaneous delivery**						
Working hours	1201063	1647	1202710	1.37		
Non-working hours	2554815	3542	2558357	1.38	1.01	0.95 to 1.07
**Instrumental delivery**						
Working hours	256279	173	256452	0.67		
Non-working hours	485085	340	485425	0.70	1.04	0.87 to 1.25
**Emergency caesarean**						
Working hours	310092	1126	311218	3.62		
Non-working hours	553239	1735	554974	3.13	0.86	0.8 to 0.93
**Planned caesarean**						
Working hours	552917	200	553117	0.36		
Non-working hours	29791	82	29873	2.74	7.61	5.86 to 9.8

* Per 1000 live births

Adjusted odds of all-cause neonatal mortality by method of birth

After adjustment, the 'protective' effect of birth in non-working hours for emergency caesareans, seen in the crude rates, was no longer evident. Odds ratios for spontaneous, instrumental and emergency caesarean deliveries were not significantly different from 1 (Table 3).

Table 3. Unadjusted and adjusted odds ratios for all-cause neonatal mortality during non-working hours versus working hours by delivery type

	Odds ratio for neonatal mortality during non-working hours compared with working hours			
	Unadjusted OR	95% CI	Adjusted OR*	95% CI
**All-cause neonatal mortality**				
Spontaneous delivery	1.01	0.95 to 1.07	0.99	0.93 to 1.06
Instrumental delivery	1.04	0.87 to 1.25	1.11	0.91 to 1.34
Emergency caesarean	0.86	0.80 to 0.93	1.06	0.98 to 1.14

* Adjusted for baby's sex, gestational age, birthweight, mode of onset of labour, geographical region, NHS trust, baby's ethnicity, baby's date of birth, maternal age and maternal parity, as well as yearly harmonic terms for day of year of the birth.

For peer review only - http://bmjopen.bmj.com/site/about/guidelines.xhtml

For peer review only

Obstetric risk factor characteristics for emergency caesareans without labour

We compared obstetric risk factor characteristics for emergency caesareans without labour during working and non-working hours (supplementary materials) (Table 4).

Table 4. Obstetric characteristics and risk factors for emergency caesareans without labour by working and non-working hours

Characteristics and risk factors	Level	Working hours	%	Non-working hours	%	*p*-value	SMD
N		60,202		71,700			
Baby's sex	F	28,505	47.3	33,670	47.0	0.160	0.008
	M	31,697	52.7	38,030	53.0		
Gestational age	22 to 28 weeks	1,127	1.9	1,439	2.0	<0.001	0.034
	28 to 32 weeks	4,182	6.9	4,926	6.9		
	32 to 37 weeks	14,368	23.9	17,167	23.9		
	37 to 42 weeks	38,228	63.5	45,008	62.8		
	42+ weeks	2,062	3.4	2,880	4.0		
	Missing	235	0.4	280	0.4		
Birthweight	Under 2,500	16,653	27.7	20,629	28.8	<0.001	0.054
	2,500-2,999	11,214	18.6	14,367	20.0		
	3,000-3,499	15,229	25.3	17,638	24.6		
	3,500-3,999	10,759	17.9	12,083	16.9		
	4,000 and over	5,156	8.6	5,691	7.9		
	Missing or unfeasible	1,191	2.0	1,292	1.8		
Maternal age	Under 20	1,899	3.2	2,697	3.8	<0.001	0.060
	20-24	8,229	13.7	10,529	14.7		
	25-29	14,603	24.3	18,051	25.2		
	30-34	18,359	30.5	21,420	29.9		
	35-39	12,920	21.5	14,444	20.1		
	Over 40	4,192	7.0	4,559	6.4		
Parity	Nulliparous	22,316	37.1	29,730	41.5	<0.001	0.091
	Moderate: 1 to 4	35,249	8.6	38,885	54.2		
	High: 5 to 9	2,511	4.2	2,940	4.1		
	Very high: 10 to 14	111	0.2	132	0.2		
	Extreme, unfeasible or missing	X	0.0	X	0.0		
Ethnicity	White British	35,770	59.4	41,519	57.9	<0.001	0.041
	White Irish	427	0.7	537	0.7		
	Any other White background	3,946	6.6	4,714	6.6		
	Mixed White & Black Caribbean	581	1.0	765	1.1		

For peer review only - http://bmjopen.bmj.com/site/about/guidelines.xhtml

	Mixed White & Black African	427	0.7	491	0.7		
	Mixed White & Asian	670	1.1	814	1.1		
	Any other Mixed background	1,064	1.8	1,365	1.9		
	Asian Indian	2,349	3.9	3,056	4.3		
	Asian Pakistani	2,910	4.8	3,706	5.2		
	Asian Bangladeshi	1,157	1.9	1,500	2.1		
	Any other Asian background	1,171	1.9	1,352	1.9		
	Black Caribbean	772	1.3	991	1.4		
	Black African	3,377	5.6	4,262	5.9		
	Any other Black background	609	1.0	737	1.0		
	Chinese	239	0.4	275	0.4		
	Any other ethnic group	1,378	2.3	1,698	2.4		
	Not Known	3,355	5.6	3,918	5.5		
Region	North East	2,053	3.4	2,405	3.4	<0.001	0.080
	North West	10,040	16.7	12,628	17.6		
	Yorkshire/Humber	4,509	7.5	5,325	7.4		
	East Midlands	2,974	4.9	3,082	4.3		
	West Midlands	8,753	14.5	12,053	16.8		
	East of England	6,423	10.7	7,572	10.6		
	London	12,590	20.9	14,129	19.7		
	South East Coast	4,254	7.1	5,047	7.0		
	South West	3,589	6	4,059	5.7		
	South Central	5,017	8.3	5,400	7.5		
Marital status at birth registration	Joint registration different address	5,524	9.2	7,082	9.9	<0.001	0.045
	Joint registration same address	16,280	27	20,222	28.2		
	Sole registration	3,749	6.2	4,702	6.6		
	Within marriage	34,649	57.6	39,694	55.4		
Mother's place of birth	United Kingdom	42,735	71	50,128	69.9	<0.001	0.028
	Other Europe	4,070	6.8	4,997	7.0		
	Africa	5,022	8.3	6,242	8.7		
	The Americas and the Caribbean	1,096	1.8	1,258	1.8		
	Asia and Middle East	6,955	11.6	8,733	12.2		
	Oceania, Antarctica and other	298	0.5	304	0.4		
	Missing	26	0.0	38	0.1		
Previous caesarean	Yes	19,030	31.6	18,119	25.3	<0.001	0.141

For peer review only

Hypertension	Yes	942	1.6	956	1.3	<0.001	0.019
Preeclampsia	Yes	6,902	11.5	8,391	11.7	0.181	0.007
Eclampsia	Yes	294	0.5	352	0.5	0.978	<0.001
Diabetes	Yes	1,600	2.7	1,463	2.0	<0.001	0.041
Gestational diabetes	Yes	3,333	5.5	3,401	4.7	<0.001	0.036
Malpresentation of foetus	Yes	9,605	16	10,639	14.8	<0.001	0.031
Maternal care for disproportion	Yes	104	0.2	155	0.2	0.087	0.010
Maternal care for abnormality of pelvic organs	Yes	17,269	28.7	16,217	22.6	<0.001	0.139
Polyhydramnios	Yes	1,301	2.2	1,377	1.9	0.002	0.017
Oligohydramnios	Yes	2,014	3.3	1,973	2.8	<0.001	0.035
Premature rupture of membranes	Yes	5,874	9.8	8,560	11.9	<0.001	0.070
Placental disorders	Yes	1,375	2.3	1,577	2.2	0.310	0.006
Placenta praevia	Yes	3,374	5.6	3,436	4.8	<0.001	0.037
Premature separation of placenta	Yes	1,831	3	3,305	4.6	<0.001	0.082
Antepartum haemorrhage	Yes	2,178	3.6	3,327	4.6	<0.001	0.051
Obstructed labour	Yes	3,280	5.4	4,502	6.3	<0.001	0.035
Intrapartum haemorrhage	Yes	410	0.7	570	0.8	0.018	0.013
Umbilical cord complications	Yes	1,166	1.9	1,864	2.6	<0.001	0.045

SMD = standardised mean difference; X = suppressed due to count(s) below 10

Point estimates from final fully adjusted model

Table 5. Odds ratios for neonatal mortality attributed to anoxia, asphyxia or trauma among emergency caesarean births without labour from 2005 to 2014

	Odds ratio	95% CI lower	95% CI upper
Spline of baby's date of birth	1.415	0.586	3.416
Spline of baby's date of birth squared	0.611	0.386	0.967
Sine term related to day-in-year	0.994	0.833	1.185
Cosine term related to day-in-year	1.031	0.867	1.226
Clocks go forward*	0	NA	NA
Clocks go back*	0	NA	NA
Weekday daytime	Ref		
Weekday night-time	1.558	1.152	2.106
Weekend/holiday daytime	0.954	0.626	1.456
Weekend/holiday night-time	1.753	1.242	2.473

For peer review only - http://bmjopen.bmj.com/site/about/guidelines.xhtml

For peer review only

Gestational age 22 to 28 weeks	0.608	0.212	1.747
Gestational age 28 to 32 weeks	0.775	0.417	1.440
Gestational age 32 to 37 weeks	1.109	0.773	1.590
Gestational age 37 to 42 weeks	Ref		
Gestational age 42 weeks and over	0.348	0.128	0.948
Gestational age missing	0.825	0.113	6.034
Birth weight under 2,500	0.981	0.623	1.545
Birth weight 2,500 to 2,999	1.367	0.931	2.008
Birth weight 3,000 to 3,499	Ref		
Birth weight 3,500 to 3,999	1.136	0.744	1.733
Birth weight 4,000 and over	1.353	0.809	2.261
Birth weight missing or unfeasible	2.774	1.395	5.517
North East	1.349	0.613	2.967
North West	1.090	0.658	1.807
Yorkshire/Humber	2.132	1.244	3.653
East Midlands	1.812	0.884	3.716
West Midlands	1.343	0.804	2.244
East of England	1.322	0.761	2.295
London	Ref		
South East Coast	1.121	0.577	2.178
South Central	1.700	0.967	2.990
South West	1.619	0.880	2.978
Spline of age of mother	0.536	0.161	1.787
Spline of age of mother squared	0.175	0.022	1.371
Nulliparous	1.184	0.906	1.547
Multiparous	Ref		
Parity missing*	0	NA	NA
Placental abruption	4.526	3.279	6.248
Maternal treatment for pelvic abnormality	0.357	0.229	0.558
Malpresentation	0.279	0.155	0.500
Preeclampsia	0.306	0.160	0.585
Postpartum haemorrhage	1.704	1.287	2.257
Antepartum haemorrhage	2.282	1.547	3.367

* No deaths; Ref = reference category; CI = confidence interval

For peer review only

Neonatal deaths attributed to anoxia, asphyxia or trauma by year

The number of births each year by emergency caesarean without labour rose from 2005 to 2014, with a larger rise in births during non-working hours than in working hours. Applying the number needed to harm of 1258 to those births during weeknights and 933 to those births during weekend/holiday nights gives a number of annual deaths potentially associated with birth during non-working hours ranging from 3.2 deaths in 2005 to 6.2 deaths in 2014 (Table 6).

Table 6. Number of deaths per year attributable to anoxia, asphyxia or trauma potentially associated with birth during non-working hours, among emergency caesarean deliveries without labour

Weekday daytime	Weekday night-time (NNH = 1258)	*Weekend/holiday daytime (NNH = 15,373)**	Weekend/holiday night-time (NNH = 933)

Birth year	N births	N births	Excess deaths**	*N births*	*Excess deaths*	N births	Excess deaths	Total excess deaths
2005	4416	2317	1.8	*1435*	*0.1*	1252	1.3	3.2
2006	4650	2448	1.9	*1426*	*0.1*	1273	1.4	3.3
2007	4623	2378	1.9	*1382*	*0.1*	1223	1.3	3.2
2008	4716	2493	2.0	*1535*	*0.1*	1277	1.4	3.4
2009	6020	3505	2.8	*2020*	*0.1*	1873	2.0	4.8
2010	6450	3610	2.9	*2087*	*0.1*	1931	2.1	4.9
2011	6855	3920	3.1	*2385*	*0.2*	2159	2.3	5.4
2012	7336	4204	3.3	*2581*	*0.2*	2324	2.5	5.8
2013	7455	4200	3.3	*2509*	*0.2*	2330	2.5	5.8
2014	7681	4483	3.6	*2708*	*0.2*	2432	2.6	6.2
Total			26.7		NA		19.4	46.0

* Not included in total as the association of birth during weekend and holiday daytimes with neonatal mortality attributed to anoxia, asphyxia or trauma was not significant

** N births / NNH (number needed to harm)

For peer review only

For peer review only - http://bmjopen.bmj.com/site/about/guidelines.xhtml

The RECORD statement – checklist of items, extended from the STROBE statement, that should be reported in observational studies using routinely collected health data.

	Item No.	STROBE items	Location in manuscript where items are reported	RECORD items	Location in manuscript where items are reported
**Title and abstract**					
	1	(a) Indicate the study's design with a commonly used term in the title or the abstract (b) Provide in the abstract an informative and balanced summary of what was done and what was found	Title and abstract	RECORD 1.1: The type of data used should be specified in the title or abstract. When possible, the name of the databases used should be included.<br><br>RECORD 1.2: If applicable, the geographic region and timeframe within which the study took place should be reported in the title or abstract.<br><br>RECORD 1.3: If linkage between databases was conducted for the study, this should be clearly stated in the title or abstract.	1.1 Abstract, manuscript p2: "Retrospective cohort linking birth registration, birth notification and hospital episode data"<br><br>1.2 Abstract, p2: "liveborn singleton births from 2005 to 2014 in NHS maternity units in England"<br><br>1.3 Abstract, p2: "Retrospective cohort linking birth registration, birth notification and hospital episode data"
**Introduction**					
Background rationale	2	Explain the scientific background and rationale for the investigation being reported	Introduction, p2-3		
Objectives	3	State specific objectives, including any prespecified hypotheses	Introduction, p2-3.		
**Methods**					
Study Design	4	Present key elements of study design early in the paper	Methods, p3-5: Data, Selection of Data for Analysis, and Statistical Analysis		

For peer review only

Setting	5	Describe the setting, locations, and relevant dates, including periods of recruitment, exposure, follow-up, and data collection	Methods, p3-4: Selection of Data for Analysis, Data		
Participants	6	*(a) Cohort study* - Give the eligibility criteria, and the sources and methods of selection of participants. Describe methods of follow-up<br>*Case-control study* - Give the eligibility criteria, and the sources and methods of case ascertainment and control selection. Give the rationale for the choice of cases and controls<br>*Cross-sectional study* - Give the eligibility criteria, and the sources and methods of selection of participants<br><br>*(b) Cohort study* - For matched studies, give matching criteria and number of exposed and unexposed<br>*Case-control study* - For matched studies, give matching criteria and the number of controls per case	Methods, p3 & fig: Data and Figure 1	RECORD 6.1: The methods of study population selection (such as codes or algorithms used to identify subjects) should be listed in detail. If this is not possible, an explanation should be provided.<br><br>RECORD 6.2: Any validation studies of the codes or algorithms used to select the population should be referenced. If validation was conducted for this study and not published elsewhere, detailed methods and results should be provided.<br><br>RECORD 6.3: If the study involved linkage of databases, consider use of a flow diagram or other graphical display to demonstrate the data linkage process, including the number of individuals with linked data at each stage.	6.1 Methods, p3: Selection of Data for Analysis, Data<br><br>6.2 References (p19-21) to Macfarlane et al 2019). Martin et al (2018), Dattani et al (2018) and Harper et al (2018)<br><br>6.3 No linkage in this study itself, and linkage well demonstrated in references cited in 6.2 above (p19-21).
Variables	7	Clearly define all outcomes, exposures, predictors, potential confounders, and effect modifiers. Give diagnostic criteria, if applicable.		RECORD 7.1: A complete list of codes and algorithms used to classify exposures, outcomes, confounders, and effect modifiers should be provided. If these cannot be reported, an explanation should be provided.	Supplementary material, p1-2
Data sources/ measurement	8	For each variable of interest, give sources of data and details of methods of assessment (measurement).	Methods, p3-5: Statistical Analysis		

For peer review only

		Describe comparability of assessment methods if there is more than one group			
Bias	9	Describe any efforts to address potential sources of bias	Methods, p3-5: Statistical analysis		
Study size	10	Explain how the study size was arrived at	Methods, p3: Selection of data for analysis, Fig. 1		
Quantitative variables	11	Explain how quantitative variables were handled in the analyses. If applicable, describe which groupings were chosen, and why	Methods, p3-5: Statistical Analysis		
Statistical methods	12	(a) Describe all statistical methods, including those used to control for confounding<br>(b) Describe any methods used to examine subgroups and interactions<br>(c) Explain how missing data were addressed<br>(d) *Cohort study* - If applicable, explain how loss to follow-up was addressed<br>*Case-control study* - If applicable, explain how matching of cases and controls was addressed<br>*Cross-sectional study* - If applicable, describe analytical methods taking account of sampling strategy<br>(e) Describe any sensitivity analyses	Methods, p3-5: Statistical Analysis		
Data access and cleaning methods		..		RECORD 12.1: Authors should describe the extent to which the investigators had access to the database population used to create the study population.	12.1 Methods, p3: Data

For peer review only

				RECORD 12.2: Authors should provide information on the data cleaning methods used in the study.	12.2 This issue was addressed at an earlier stage of the project, see DOIs 10.3310/hsdr07180 and 10.1136/bmjopen-2017-017898
Linkage		..		RECORD 12.3: State whether the study included person-level, institutional-level, or other data linkage across two or more databases. The methods of linkage and methods of linkage quality evaluation should be provided.	12.3 Methods, p3: Data
**Results**					
Participants	13	(a) Report the numbers of individuals at each stage of the study (*e.g.*, numbers potentially eligible, examined for eligibility, confirmed eligible, included in the study, completing follow-up, and analysed)<br>(b) Give reasons for non-participation at each stage.<br>(c) Consider use of a flow diagram	Methods: Data and Figure 1	RECORD 13.1: Describe in detail the selection of the persons included in the study (*i.e.,* study population selection) including filtering based on data quality, data availability and linkage. The selection of included persons can be described in the text and/or by means of the study flow diagram.	Methods, p3: Selection of data for analysis and Figure 1
Descriptive data	14	(a) Give characteristics of study participants (*e.g.*, demographic, clinical, social) and information on exposures and potential confounders<br>(b) Indicate the number of participants with missing data for each variable of interest<br>(c) *Cohort study* - summarise follow-up time (*e.g.*, average and total amount)	Results: Table 2 (p8); Online supplement table 4 (p4-6)		

For peer review only

Outcome data	15	*Cohort study* - Report numbers of outcome events or summary measures over time<br>*Case-control study* - Report numbers in each exposure category, or summary measures of exposure<br>*Cross-sectional study* - Report numbers of outcome events or summary measures	Results, table 3 (p10)		
Main results	16	(a) Give unadjusted estimates and, if applicable, confounder-adjusted estimates and their precision (e.g., 95% confidence interval). Make clear which confounders were adjusted for and why they were included<br>(b) Report category boundaries when continuous variables were categorized<br>(c) If relevant, consider translating estimates of relative risk into absolute risk for a meaningful time period	Results: Tables 3-8 and section Absolute risks from emergency caesarean without labour during non-working hours (pp10-13)		
Other analyses	17	Report other analyses done—e.g., analyses of subgroups and interactions, and sensitivity analyses	Results, p12: Timing within non-working hours		
**Discussion**					
Key results	18	Summarise key results with reference to study objectives	Discussion, p13-14		
Limitations	19	Discuss limitations of the study, taking into account sources of potential bias or imprecision. Discuss both direction and magnitude of any potential bias	Discussion, p15-16	RECORD 19.1: Discuss the implications of using data that were not created or collected to answer the specific research question(s). Include discussion of misclassification bias, unmeasured confounding, missing data, and changing eligibility over	Discussion, p15-16

For peer review only

				time, as they pertain to the study being reported.	
Interpretation	20	Give a cautious overall interpretation of results considering objectives, limitations, multiplicity of analyses, results from similar studies, and other relevant evidence	Discussion, p16		
Generalisability	21	Discuss the generalisability (external validity) of the study results	Discussion, p15		
**Other Information**					
Funding	22	Give the source of funding and the role of the funders for the present study and, if applicable, for the original study on which the present article is based	Funding statement, p19		
Accessibility of protocol, raw data, and programming code		..		RECORD 22.1: Authors should provide information on how to access any supplemental information such as the study protocol, raw data, or programming code.	Supplementary information is provided in an online supplement. Complete open data sharing is not possible because of restriction of access to personal data used in this study. Manuscript p18

*Reference: Benchimol EI, Smeeth L, Guttmann A, Harron K, Moher D, Petersen I, Sørensen HT, von Elm E, Langan SM, the RECORD Working Committee. The REporting of studies Conducted using Observational Routinely-collected health Data (RECORD) Statement. *PLoS Medicine* 2015; in press.

*Checklist is protected under Creative Commons Attribution (CC BY) license.

For peer review only - http://bmjopen.bmj.com/site/about/guidelines.xhtml

BMJ Open

Neonatal mortality in NHS maternity units by timing and mode of birth: a retrospective linked cohort study

Journal:	*BMJ Open*
Manuscript ID	bmjopen-2022-067630.R1
Article Type:	Original research
Date Submitted by the Author:	13-Apr-2023
Complete List of Authors:	Carty, Lucy; City University of London, Midwifery<br>Grollman, Christopher; City University of London, Midwifery<br>Plachcinski, Rachel; City University of London, Midwifery<br>Cortina Borja, Mario; University College London, UCL Institute of Child Health<br>Macfarlane, Alison; City University of London, Midwifery
**Primary Subject Heading**:	Epidemiology
Secondary Subject Heading:	Health services research, Obstetrics and gynaecology
Keywords:	NEONATOLOGY, OBSTETRICS, HEALTH SERVICES ADMINISTRATION & MANAGEMENT

SCHOLARONE™
Manuscripts

I, the Submitting Author has the right to grant and does grant on behalf of all authors of the Work (as defined in the below author licence), an exclusive licence and/or a non-exclusive licence for contributions from authors who are: i) UK Crown employees; ii) where BMJ has agreed a CC-BY licence shall apply, and/or iii) in accordance with the terms applicable for US Federal Government officers or employees acting as part of their official duties; on a worldwide, perpetual, irrevocable, royalty-free basis to BMJ Publishing Group Ltd ("BMJ") its licensees and where the relevant Journal is co-owned by BMJ to the co-owners of the Journal, to publish the Work in this journal and any other BMJ products and to exploit all rights, as set out in our licence.

The Submitting Author accepts and understands that any supply made under these terms is made by BMJ to the Submitting Author unless you are acting as an employee on behalf of your employer or a postgraduate student of an affiliated institution which is paying any applicable article publishing charge ("APC") for Open Access articles. Where the Submitting Author wishes to make the Work available on an Open Access basis (and intends to pay the relevant APC), the terms of reuse of such Open Access shall be governed by a Creative Commons licence – details of these licences and which Creative Commons licence will apply to this Work are set out in our licence referred to above.

Other than as permitted in any relevant BMJ Author's Self Archiving Policies, I confirm this Work has not been accepted for publication elsewhere, is not being considered for publication elsewhere and does not duplicate material already published. I confirm all authors consent to publication of this Work and authorise the granting of this licence.

For peer review only

Neonatal mortality in NHS maternity units by timing and mode of birth: a retrospective linked cohort study

Lucy Carty* (0000-0003-2849-3145)

Chris Grollman* (0000-0002-6950-1837)

Rachel Plachcinski (0000-0001-9908-0773)

Mario Cortina-Borja (0000-0003-0627-2624)

Alison Macfarlane (0000-0003-0977-7214)

* Joint first authors

Lucy Carty, City, University of London (lucy.carty@city.ac.uk, ORCID: 0000-0003-2849-3145)

Christopher Grollman, City, University of London (christopher.grollman@city.ac.uk, ORCID: 0000-0002-6950-1837)

Mario Cortina-Borja, University College London (m.cortina@ucl.ac.uk, ORCID: 0000-0003-0627-2624)

Rachel Plachcinski, City, University of London (rachel.plachcinski@city.ac.uk, ORCID: 0000-0001-9908-0773)

Alison Macfarlane, City, University of London (a.j.macfarlane@city.ac.uk, ORCID: 0000-0003-0977-7214, corresponding author)

For peer review only

Word count for the main text (excluding the abstract, references, tables, boxes, or figures): 5906

Structured abstract

Objectives To compare neonatal mortality in English hospitals by time of day and day of the week according to care pathway.

Design Retrospective cohort linking birth registration, birth notification and hospital episode data

Setting NHS hospitals in England

Participants 6,054,536 liveborn singleton births from 2005 to 2014 in NHS maternity units in England

Main outcome measures Neonatal mortality

Results

After adjustment for confounders, there was no significant difference in the odds of neonatal mortality attributed to asphyxia, anoxia or trauma outside of working hours compared with working hours for spontaneous births or instrumental births. Stratification of emergency caesareans by onset of labour showed no difference in mortality by birth timing for emergency caesareans with spontaneous or induced onset of labour. Higher odds of neonatal mortality attributed to asphyxia, anoxia or trauma out of hours for emergency caesareans without labour translated to a small absolute difference in mortality risk.

For peer review only - http://bmjopen.bmj.com/site/about/guidelines.xhtml

Conclusions

The apparent ‘weekend effect’ may result from deaths among the relatively small numbers of babies who were coded as born by emergency caesarean section without labour outside normal working hours. Further research should investigate the potential contribution of care-seeking and community-based factors as well as the adequacy of staffing for managing these relatively unusual emergencies.

Strengths and limitations

- This analysis used a large linked dataset bringing together data for over 6 million births over ten years which enabled stratification by mode of onset of labour and mode of birth, and by time of day as well as day of birth.
- Stillbirths: Stillbirths were excluded as around 90 per cent of fetal deaths occur before the onset of labour so the timing of death is unknown.
- Adjustment for covariates: The large number of variables available made it possible to adjust for covariates including obstetric risk factors and the seasonal and temporal nature of birth.
- The subdivision of caesarean births by timing of decision into elective and emergency used in the UK has enabled the identification of a group which would not have been visible using the subdivision of caesareans into ‘before labour’ and ‘in labour’ used in many other countries.
- Variables were all derived from anonymised hospital administrative data, so there were no data available about events in the community before admission to hospital or after discharge.

Introduction

Since 2001, a considerable number of analyses have shown higher mortality rates among patients admitted to hospital at weekends compared with weekdays. There has been considerable debate about how to interpret these rates, with differences in quality of care and differences in case mix being frequently cited;[1, 2] concerns about a ‘weekend effect’ led to a ‘7-day services’ policy for the NHS in England in 2015.[3] A systematic review looking at the need to increase specialist intensity at weekends for patients undergoing emergency hospital admission, published in 2021, concluded that there was unlikely to be a single cause for the weekend effect. It pointed to the importance of case mix and concluded that the effect is unlikely to be an indicator of quality of hospital care.[4, 5]

In the perinatal field, studies have investigated pregnancy outcomes by day of the week, time of the year and time of day, particularly from the 1970s onwards. Analyses of perinatal mortality in England and Wales among births in the years 1970–76[6-8] and 1979–96[9] showed higher mortality rates among babies born at weekends, but could only estimate crude rates, meaning no conclusions could be drawn. Studies which were able to adjust for confounding produced inconsistent results. A study in Canada found slightly higher crude rates of stillbirths and neonatal deaths among births at weekends, but the difference disappeared after adjustment for gestational age,[10] and a study in

For peer review only - http://bmjopen.bmj.com/site/about/guidelines.xhtml

Australia found no difference after adjustment for birthweight.[11] An analysis by day of the week in England published in 2015 concluded that perinatal mortality was highest at the weekend.[12] Some studies showed seasonal or short-term variation. Analyses of data for 1993–95 from Wales[13] and Scotland[14], with relatively small numbers of deaths, suggested a possible association of mortality with the rotation of junior doctors to new posts in August. An analysis for England and Wales for 1979–96 showed seasonal variation, with higher perinatal mortality rates in winter than in summer.[9] Studies investigating time of day from Switzerland,[15, 16] Sweden[17, 18] and California[19] found higher mortality at night, although they reported varied risks in terms of type of perinatal death and time of night. An analysis of neonatal deaths at term in Scotland from 1985-2004 compared deaths among babies born from 9.00-17.00 on Mondays to Fridays to babies born outside these hours and days. It found higher rates of deaths ascribed to intrapartum anoxia among babies born 'out of hours' but no differences for other causes of death.[20]

The 2021 systematic review [4] recommended that further work should focus on underlying mechanisms and examine care processes in both hospital and the community. Such work has the potential to draw out the contributions of both case-mix and staffing questions to observed higher mortality. In this analysis, we have analysed neonatal mortality of babies born during and outside of working hours in English hospitals according to mode of birth and onset of labour.

Methods

Data

This study uses linked data from birth registration, birth notification and maternity hospital episode statistics.

Information about births in England and Wales is recorded in several systems. Sociodemographic information is recorded at birth registration. Further information, including gestational age and time of birth, is recorded at birth notification when each baby is issued with a National Health Service (NHS) number. Information about care at birth in NHS hospitals in England is recorded in Maternity Hospital Episode Statistics (HES) within the mother-based HES deliveries file. Further information about the health of the baby and level of care required after birth is recorded in the baby-based HES baby file.

Following a series of pilot projects in collaboration with City, University of London, the Office for National Statistics (ONS) now routinely links birth registration and birth notification data. City, University of London has linked these data to HES and also to corresponding data for Wales to form the City Birth Cohort.[21] Authors had full access to the data from these previous efforts, and this study did not itself include further linkage.

Selection of data for analysis

This study uses a source dataset derived from the City Birth Cohort and consisting of all 6,054,536 singleton births occurring in NHS maternity units in England from 2005 to 2014 and with good links to HES. Derivation and analysis of linkage bias for this cohort has been described elsewhere.[22-24] In summary, it was possible to link over 94 per cent of birth registration linked to notification records to HES delivery and birth records. The linkage rate increased over time as the quality of Maternity. of Maternity HES improved.

From this population, we removed live births occurring before 22 weeks of gestational age and births registered as stillborn. Nearly 90 per cent of stillbirths in England are recorded as antepartum,

For peer review only

with fetal death occurring before the onset of labour.[25] For the remaining stillbirths, we attempted to identify those where death occurred intrapartum but found that the timing of stillbirth was poorly recorded in both the CMACE data and the ONS birth registration data. As a result, for a substantial proportion of the relatively small number of records we could not determine whether the stillbirth was antepartum or intrapartum, so we chose to exclude all stillbirths from our outcome indicator.

By contrast, virtually all neonatal deaths are identified unambiguously so we have higher confidence in the completeness of that population. We used the ONS' modified Wigglesworth classification system[26] to classify neonatal deaths and removed deaths attributed to congenital anomalies. To derive the analysis population for modelling, we further removed births for which the time of birth was missing. We determined that recorded time of birth was subject to heaping on 5-minute intervals but that there were no other important accumulation points.

Statistical analysis

We used the mean and standard deviation to summarise continuous variables and *t*-test for comparisons between groups. We used percentages to summarise categorical variables and χ^2 test for comparisons between groups.

The primary outcome was cause-specific neonatal mortality attributed to asphyxia, anoxia or trauma using the ONS's modified Wigglesworth categories,[26, 27] as this is the category of death most likely to be affected by quality of care and staffing factors relevant to the 'weekend effect'; deaths attributed to intrapartum anoxia were responsible for elevated out-of-hours mortality in a study of over 1 million singleton term live births in Scotland between 1985 and 2004.[20] We also modelled all-cause neonatal mortality unattributed to congenital anomaly (Table 1).

We used an 8-day categorisation for day of birth which included Monday to Sunday and public holidays. Time of birth was categorised into daytime hours from 07:00 to 19:00 and night-time hours. A combination of day of birth and time of birth was used to classify births as occurring during 'working hours', defined as weekday daytime hours, and 'out of hours', defined as all other times of the week and including all nights, weekends and holidays. In breaking down out-of-hours further, the hours from midnight to 7am on a Monday were classified as a weekend/holiday night to reflect the fact that people giving birth in that period would have had access to weekend staffing only in the preceding 48 hours.

The dataset was stratified by mode of birth and later analyses by mode of onset of labour. To determine the mode of birth we used the Office of Population Census and Surveys (OPCS) procedure codes recorded in the standard HES record, supplemented by the HES 'maternity tail' variable DELMETH, as previously described.[22]

We used the HES maternity tail variable DELONSET to determine the mode of onset of labour. Models were constructed for each mode of birth with onset of labour as a covariate.

We identified candidate covariates and confounders fitting univariable and multivariable logistic regression models and used a combination of forwards and backwards selection to determine inclusion of covariates in the models. Models were compared using the likelihood ratio test and the Bayesian information criterion (BIC).[28]

Long-term trends, and mother's age were characterised using natural cubic splines with the amount of smoothing chosen by minimising BIC as a function of degrees of freedom. Day of birth in the year was modelled using yearly and semestral harmonic terms. Binary contrasts referred to sex of the

For peer review only - http://bmjopen.bmj.com/site/about/guidelines.xhtml

baby and changes between Greenwich Mean Time (GMT) and British Summer Time (BST). Birth attendants notifying births are instructed to notify gestational age in completed weeks from last menstrual period but some may have used gestational age as recorded by ultrasound as this has become routine. Birthweight was measured in grams and categorised into five levels for modelling. Ethnicity as recorded at birth notification was coded using 17 categories based on the ethnicity question in the 2001 Census of England and Wales. Parity was defined as nulliparous or multiparous by combining information from ONS and HES and by reviewing linked HES records' Mother IDs to determine whether there had been previous births.[29]

We accounted for geographic variation by adjusting for the former Strategic Health Authority (SHA) Region of England where the birth took place. Random effects terms were included in the models to allow for clustering of providers within the NHS trust where the birth took place. As the constituent hospitals making up some trusts varied over the ten-year time period, maternity units were allocated to a single trust (the 'Assigned Trust') for the entire period, even if they were not part of this Trust for the whole time, as described in the project report.[29] At the suggestion of a reviewer we checked whether the inclusion of trust could have adjusted away community effects that may have contributed to adverse outcomes. A sensitivity analysis excluding trust and using a GLM model without random effects showed no effect on the estimate for our birth timing variable. The risk of cause-specific and all-cause neonatal mortality was modelled fitting mixed-effects logistic regression models including a random effects term on the intercept to account for unobserved heterogeneity between Assigned Trusts.

During exploratory analyses we found associations between missing information for some variables and neonatal mortality. We also found non-random missingness patterns for key variables including gestational age and birthweight which was not suitable for imputation. For this reason, predictors were included in the models as categorical variables with a category for missing information. Data completeness, particularly for HES, has improved over time. We also observed this pattern in birthweight and gestational age data recorded at birth notification and registration; the percentage of births for which either birthweight or gestational age were missing was under 1%, well below the extent to which data were missing from HES. For around 15% of births mode of onset of labour was unknown. They were included in the analyses that did not stratify by mode of onset, and we included them in models as an unknown category rather than considering them as missing data.

Following a suggestion from our PPI group, we calculated an absolute measure of risk – the number needed to harm (NNH) – as well as the relative measure presented by the odds ratio. The NNH was calculated as:

$$NNH = ((PEER \times (OR - 1)) + 1) \div (PEER \times (OR - 1) \times (1 - PEER))$$

where the patient expected event rate (PEER) was the rate of neonatal death attributed to asphyxia, anoxia or trauma in the unexposed timing category, and the odds ratio (OR) was that from the adjusted logistic regression model.

Coding of all variables is shown in supplementary table 1. All analyses were performed in the ONS Secure Research Service with R version 3.6.1.

Patient and Public Involvement

Public involvement and engagement (PI&E) have been at the core of the work undertaken to develop and analyse the City Birth Cohort using a documented 'three-tier' approach [25]. Two

For peer review only - http://bmjopen.bmj.com/site/about/guidelines.xhtml

service user representatives were involved in the earlier NIHR-funded research from the outset as co-applicants. At the design stage, they contributed to the funding application and once funding was obtained they led the public engagement and involvement aspects of the project and helped to direct the focus of the research. In the ESRC-funded research described here, service ser representatives from Maternity Voices Partnerships (MVPs) became involved. The PI&E strategy was based on the same three-tier model [25] For this particular project our PI&E lead presented previous findings and our new plans at a national event for Maternity Voices Partnership lay members in order to collect the views of a wider constituency of parents, and also invited expressions of interest in joining a new PI&E advisory group. Three people (White British, Black British and Asian British) joined the group. Another MVP member joined our external advisory group at a later date. The PI&E group has met with the PI&E lead and data analyst regularly throughout the project to discuss progress and findings, including moving online during the COVID-19 pandemic. They have raised issues, such as the level of detail available on ethnicity, which have fed into the models, and stressed the importance of contextualising messages for both lay and clinical audiences. Group members have worked with the PI&E lead and data analyst to develop guides to publicly available datasets and reports on five topics of particular concern to them: ethnicity in maternity statistics, miscarriage, birth interventions, prematurity and smoking in pregnancy. They also presented their learning, experiences and recommendations for future data intensive research at a dissemination event for parents and service user representatives. A second, multidisciplinary, event is planned in Spring 2023 and we will also share our results at future MVP conferences.

Figure 1: Population

[figure 1]

Results

Numbers and causes of deaths in the population

The source dataset consisted of 6,054,536 singleton births taking place in NHS maternity units in England between 1st January 2005 and 31st December 2014. We excluded 25,748 births recorded as stillborn (4.25 per 1000 births) and 1,782 births recorded as occurring at less than 22 weeks gestational age (0.03%).

Table 1 shows neonatal deaths of babies born alive after 22 weeks of gestation. The group of intrapartum causes – asphyxia, anoxia and trauma – caused the third highest number of neonatal deaths. The neonatal mortality rate for deaths attributed to asphyxia, anoxia or trauma was 0.25 per 1000 live births.

Table 1. Neonatal deaths by modified Wigglesworth cause of death categories

Causes of death	Neonatal deaths, *n*
**Cause arises before the onset of labour:**	
Congenital anomalies	4,070
Antepartum infections	489

Immaturity related conditions	6,178
**Cause arises during, or shortly after labour and birth:**	
Asphyxia, anoxia or trauma	1,494
**Cause arises after birth:**	
External conditions	44
Infections	237
Other specific conditions	111
Sudden infant deaths	190
**Unclassified:**	
Other conditions	239
Missing	25
**Total**	**13,077**

For peer review only

We further excluded 6,494 births recorded as infant deaths (including 4,070 neonatal deaths) attributed to congenital anomalies (0.11%), leaving a descriptive population of 6,020,512 births (Figure 1).

Characteristics of population for analysis of timing of birth

To derive the analysis population, we then excluded 55,864 births that had missing information about time of birth as we could not categorise these as exposed or unexposed. We did not find any evidence that missing time of birth was associated with either neonatal mortality (Chi-squared p = 0.75) or neonatal mortality attributed to asphyxia, anoxia or trauma (Chi-squared p = 1).

Baseline characteristics in the analysis population (N = 5,964,648) were stratified by whether births took place inside weekday working hours (weekdays 07:00 to 19:00) or outside working hours (nights, weekends and holidays) (Table 2). The proportion of births that took place outside of weekday working hours during nights, weekends or holidays was 61.0%, lower than the 65.1% of hours that were outside working hours. The standardised mean difference (SMD) was small for all characteristics (<0.10), except for mother's age, parity and previous caesareans. Among multiparous women, 32.0% of births during working hours were to people who had previously had a caesarean compared with 10.5% of those out of hours. Birth during working hoursalso included higher proportions of older mothers..

Table 2. Characteristics of births in analysis population inside and outside of weekday working hours

Characteristic	Level	Working hours	%	Out of hours	%	*p*-value	SMD
N		2,328,731		3,635,917			
Baby sex	F	1,134,931	48.7	1,766,765	48.6	0.001	0.003
	M	1,193,800	51.3	1,869,152	51.4		
Gestational age	22 to 28 weeks	6,764	0.3	12,025	0.3	<0.001	0.063
	28 to 32 weeks	15,219	0.7	21,832	0.6		
	32 to 37 weeks	115,228	4.9	175,908	4.8		
	37 to 42 weeks	2,097,955	90.1	3,236,055	89.0		

For peer review only - http://bmjopen.bmj.com/site/about/guidelines.xhtml

	42 weeks and over	76,852	3.3	163,639	4.5		
	Missing	16,713	0.7	26,458	0.7		
Birthweight	Under 2,500	129,603	5.6	204,111	5.6	<0.001	0.012
	2,500-2,999	369,786	15.9	589,999	16.2		
	3,000-3,499	848,644	36.4	1,318,645	36.3		
	3,500-3,999	694,708	29.8	1,086,195	29.9		
	4,000 and over	272,305	11.7	415,955	11.4		
	Missing or unfeasible	13,685	0.6	21,012	0.6		
Mother's age	Under 20	115,846	5.0	220,044	6.1	<0.001	0.116
	20-24	401,146	17.2	711,565	19.6		
	25-29	618,854	26.6	1,019,383	28.0		
	30-34	684,650	29.4	1,025,162	28.2		
	35-39	407,151	17.5	539,755	14.8		
	Over 40	101,084	4.3	120,008	3.3		
Parity	Nulliparous	892,405	38.3	1,594,567	43.9	<0.001	0.114
	Moderate: 1 to 4	1,367,620	58.7	1,935,522	53.2		
	High: 5 to 9	66,080	2.8	101,595	2.8		
	Very high: 10 to 14	2,409	0.1	3,905	0.1		
	Extreme/unfeasible	169	0.0	257	0.0		
	Missing	48	0.0	71	0.0		
Ethnicity	White British	1,491,685	64.1	2,319,066	63.8	<0.001	0.019
	White Irish	14,809	0.6	22,483	0.6		
	Any other White background	175,116	7.5	274,915	7.6		
	Mixed White & Black Caribbean	23,427	1.0	38,851	1.1		
	Mixed White & Black African	14,880	0.6	23,188	0.6		
	Mixed White & Asian	24,680	1.1	38,546	1.1		
	Any other Mixed background	41,207	1.8	64,505	1.8		
	Asian Indian	71,267	3.1	111,751	3.1		
	Asian Pakistani	94,864	4.1	155,558	4.3		
	Asian Bangladeshi	32,892	1.4	53,091	1.5		
	Any other Asian background	37,163	1.6	56,125	1.5		
	Black Caribbean	21,566	0.9	35,681	1.0		
	Black African	74,878	3.2	111,342	3.1		
	Any other Black background	17,003	0.7	26,458	0.7		
	Chinese	11,253	0.5	18,536	0.5		
	Any other ethnic group	48,855	2.1	74,154	2.0		
	Not Known	133,186	5.7	211,667	5.8		
Region	North East	113,142	4.9	173,066	4.8	<0.001	0.022
	North West	314,194	13.5	490,345	13.5		
	Yorkshire/Humber	228,995	9.8	373,778	10.3		

For peer review only

For peer review only - http://bmjopen.bmj.com/site/about/guidelines.xhtml

	East Midlands	173,054	7.4	273,713	7.5		
	West Midlands	254,354	10.9	405,784	11.2		
	East of England	231,783	10.0	358,103	9.8		
	London	461,053	19.8	704,290	19.4		
	South East Coast	186,082	8.0	282,432	7.8		
	South West	188,108	8.1	299,581	8.2		
	South Central	177,966	7.6	274,825	7.6		
Marital status at birth registration	Joint registration different address	215,147	9.2	366,533	10.1	<0.001	0.054
	Joint registration same address	681,512	29.3	1,111,692	30.6		
	Sole registration	134,867	5.8	227,363	6.3		
	Within marriage	1,297,198	55.7	1,930,320	53.1		
	Missing	7	0.0	9	0.0		
Mother's country of birth	United Kingdom	1,743,087	74.9	2,729,074	75.1	<0.001	0.016
	Other Europe	185,255	8.0	296,498	8.2		
	Africa	128,846	5.5	190,826	5.2		
	The Americas and the Caribbean	36,325	1.6	53,466	1.5		
	Middle East and Asia	221,835	9.5	346,122	9.5		
	Oceania and Antarctica	10,477	0.5	15,475	0.4		
	Missing	1,453	0.1	2,228	0.1		
Previous caesarean birth (multiparous women only)	No	976,973	68.0	1,826,456	89.5	<0.001	0.544
	Yes	459,188	32.0	214,645	10.5		

Crude neonatal mortality rates

Crude rates of cause-specific neonatal mortality attributed to asphyxia, anoxia or trauma were higher for births out of hours than for births during working hours (0.27 compared with 0.21 per 1000 live births, rate ratio 1.26, 95% confidence interval 1.13 to 1.40). The crude mortality rate ratio was not significantly different from 1 for spontaneous, instrumental or emergency caesarean births. The elevated rate in the overall population was driven by a large rate ratio among births recorded as planned caesareans but occurring out of hours (Table 3), an estimate which is likely to be highly biased due to misclassification, as we discuss below. Due to this issue of misclassification, we did not include planned caesareans in the modelling analyses. Results were similar for all-cause mortality, except that the crude mortality rate ratio was significantly below 1 for emergency caesarean births (0.86, 0.80 to 0.93), meaning a lower crude all-cause neonatal mortality rate for births out of hours than for births during working hours (supplementary table 2).

Table 3. Crude cause-specific neonatal mortality attributed to asphyxia, anoxia or trauma by time and day of birth, stratified by mode of birth

	Neonatal deaths	Total live births	Rate*	Rate ratio	95% CI
**Total population**					

For peer review only - http://bmjopen.bmj.com/site/about/guidelines.xhtml

For peer review only

Working hours	499	2,328,731	0.21		
Out of hours	981	3,635,917	0.27	1.26	1.13 to 1.4
**Spontaneous birth**					
Working hours	115	1,202,710	0.10		
Out of hours	264	2,558,357	0.10	1.08	0.87 to 1.35
**Instrumental birth**					
Working hours	60	256,452	0.23		
Out of hours	107	485,425	0.22	0.94	0.69 to 1.3
**Emergency caesarean**					
Working hours	301	311,218	0.97		
Out of hours	587	554,974	1.06	1.09	0.95 to 1.26
**Planned caesarean**					
Working hours	21	553,117	0.04		
Out of hours	16	29,873	0.54	14.11	7.25 to 26.94

* Per 1000 live births

Adjusted odds of neonatal mortality by type of birth

We fitted multivariable mixed-effects logistic regression models to isolate the effect of working hours from potential confounding by other factors associated with neonatal mortality attributed to asphyxia, anoxia or trauma. Our final models were adjusted for baby's sex, gestational age, birthweight, mode of onset of labour, geographical region, NHS trust, baby's ethnicity, baby's date of birth, mother's age and parity, as well as yearly harmonic terms for day of year of the birth. After adjustment, there was no significant difference in the odds of mortality out of hours compared with working hours for spontaneous or instrumental births. The odds of neonatal mortality attributed to asphyxia, anoxia or trauma for emergency caesareans was significantly higher out of hours compared with during working hours (OR, 1.21; 95% CI, 1.05 to 1.39) (Table 4). For all-cause neonatal mortality, the odds ratios were not significantly different from 1 for any mode of birth (supplementary table 3).

Table 4. Unadjusted and adjusted odds ratios for neonatal mortality attributed to asphyxia, anoxia or trauma out of hours compared with during working hours, by type of birth

	Out of hours compared with working hours			
	Unadjusted OR	95% CI	Adjusted OR*	95% CI
**Neonatal mortality attributed to asphyxia, anoxia or trauma**				
Spontaneous birth	1.08	0.87 to 1.34	1.09	0.87 to 1.35
Instrumental birth	0.94	0.69 to 1.29	0.96	0.70 to 1.32
Emergency caesarean	1.09	0.95 to 1.26	1.21	1.05 to 1.39

* Adjusted for baby's sex, gestational age, birthweight, mode of onset of labour, geographical region, NHS trust, baby's ethnicity, baby's date of birth, maternal age and maternal parity, as well as yearly harmonic terms for day of year of the birth and a natural spline for day of the study period.

Onset of labour for emergency caesarean births

We further stratified emergency caesareans by mode of onset of labour. The largest group of emergency caesareans occurred after spontaneous onset, followed by induced onset, and fewer than one fifth are emergency caesareans before the start of labour. These emergency caesarean

For peer review only - http://bmjopen.bmj.com/site/about/guidelines.xhtml

For peer review only

births without labour constituted 2.2% of all births in the dataset. The rates of neonatal mortality were highest among the group with no labour, and that is also the only group for which the rate of neonatal mortality was significantly higher out of hours compared to during working hours (Table 5).

Table 5. Crude cause-specific neonatal mortality attributed to asphyxia, anoxia or trauma by time and day of birth, among emergency caesareans births, stratified by mode of onset of labour

	Neonatal deaths	Total live births	Rate*	Rate ratio	95% CI
**Spontaneous onset**					
Working hours	103	120,791	0.85		
Out of hours	204	226,528	0.90	1.056	0.84 to 1.34
**Induced onset**					
Working hours	29	75,513	0.38		
Out of hours	71	162,353	0.44	1.139	0.75 to 1.78
**No labour**					
Working hours	86	60,202	1.43		
Out of hours	169	71,700	2.36	1.65	1.28 to 2.15

* Per 1000 live births

The odds of neonatal mortality attributed to asphyxia, anoxia or trauma for emergency caesareans without labour were two thirds higher for births out of hours compared with births during working hours, unaffected by adjustment for characteristics of the mother, baby and birth (OR, 1.66; 95% CI, 1.28 to 2.16) (Table 6). No significant difference by working hours was seen in neonatal mortality attributed to asphyxia, anoxia or trauma pre for emergency caesareans with spontaneous or induced onset.

Babies born by emergency caesarean without labour are a group at inherently high risk, so to estimate the effect of out-of-hours care we further adjusted the model for this group of births for obstetric risk factors. We compared obstetric risk factor characteristics during and out of working hours (supplementary table 4) and included in the model those that improved fit. After adjustment, there remained 48% higher odds of neonatal mortality attributed to asphyxia, anoxia or trauma out of hours compared with working hours (OR 1.48; 95% CI 1.14 to 1.92) (Table 6). The full coefficients in the fully adjusted model are presented in supplementary table 5.

Table 6. Unadjusted and adjusted odds ratios for neonatal mortality attributed to asphyxia, anoxia or trauma out of hours compared with during working hours for emergency caesareans

	Odds ratios, out of hours compared with during working hours					
Mode of onset	Unadjusted	95% CI	Adjusted for mother, baby and birth characteristics*	95% CI	Further adjusted for obstetric risk factors**	95% CI
**Spontaneous**	1.06	0.83 to 1.34	1.06	0.83 to 1.34	--	--
**Induced**	1.14	0.74 to 1.75	1.14	0.74 to 1.76	--	--
**Caesarean (no labour)**	1.65	1.27 to 2.14	1.66	1.28 to 2.16	1.48	1.14 to 1.92

* Adjusted for baby's sex, gestational age, birthweight, geographical region, NHS trust, baby's ethnicity, baby's date of birth, maternal age and maternal parity, as well as yearly harmonic terms

For peer review only - http://bmjopen.bmj.com/site/about/guidelines.xhtml

for day of year of the birth. ** Further adjusted for placental abruption, maternal care for abnormality of pelvic organs, malpresentation of the fetus, preeclampsia, postpartum haemorrhage, and antepartum haemorrhage.

Timing out of hours

To understand better the circumstances in which births were at higher risk of neonatal mortality we disaggregated the “out of hours” category to allow us to distinguish evenings from weekends and holidays. Among emergency caesarean births without labour, being born during daytime hours of 07:00 to 19:00 at the weekend or on a holiday held no greater risk of mortality attributed toasphyxia, anoxia or trauma than being born during daytime hours on a working weekday. By contrast, emergency caesarean birth without labour in the night-time held an increased risk both during weeknights (odds ratio = 1.56, 95%CI 1.15 to 2.11) and night-time at weekends and holidays (odds ratio = 1.75, 95%CI 1.24 to 2.47) (Table 7). There still was no association between mortality risk and working hours for spontaneous or instrumental birth or emergency caesareans with spontaneous/induced onset using the four-category timing variable (data not shown).

Table 7. Crude numbers and rates, and modelled odds ratios, for neonatal mortality attributed to asphyxia, anoxia or trauma out of hours compared with weekday daytime working hours for emergency caesareans without labour

	Neonatal deaths	Total live births	Crude rate*	Adjusted** odds ratio	95% CI
**Weekday daytime**	86	60202	1.43	*Ref*	*Ref*
**Weekday night-time**	86	33558	2.56	1.56	1.15 to 2.11
**Weekend/holiday daytime**	29	20068	1.45	0.95	0.63 to 1.46
**Weekend/holiday night-time**	54	18074	2.99	1.75	1.24 to 2.47

* Per 1000 live births

** Adjusted for baby’s sex, gestational age, birthweight, geographical region, NHS trust, baby’s ethnicity, baby’s date of birth, maternal age and maternal parity, as well as yearly harmonic terms for day of year of the birth, and presence of obstetric risk factors (placental abruption, maternal care for abnormality of pelvic organs, malpresentation of the fetus, preeclampsia, postpartum haemorrhage, and antepartum haemorrhage).

We further investigated whether risk of death out of hours was significantly associated with gestational age, local-area deprivation (using deciles of the Index of Multiple Deprivation) or year of birth (data not shown). We found that the only gestational age category where birth out of hours had a significant crude rate ratio for death attributed to asphyxia, anoxia or trauma compared to working hours was among term births (37 to 42 weeks). We found no evidence that the association of birth timing with mortality differed by epoch of birth (characterised in two-year epochs and in five-year epochs across the period 2005 to 2014). Lower local-area deprivation appeared to have had a small protective effect but this was not significant..

Absolute risks from emergency caesarean without labour out of hours

We calculated how many emergency caesareans without labour would have to occur out of hours to be associated with a neonatal death attributed to asphyxia, anoxia or trauma beyond those expected if all emergency caesareans without labour happened during working hours. The patient expected event rate (PEER) was taken as the rate of neonatal death attributed to asphyxia, anoxia or trauma among emergency caesareans without labour during weekday working hours, 1.43 per 1000

For peer review only - http://bmjopen.bmj.com/site/about/guidelines.xhtml

live births (Table 5), and the odds ratios from the model further adjusted for obstetric risk factors were 1.56 for weekday night-time and 1.75 for weekend/holiday night-time (Table 7). The resulting NNHs were 1258 for weekday nights and 933 for weekend/holiday nights. This means that for every 1258 births by emergency caesarean without labour born on a weekday night, there would be one additional neonatal death attributed to asphyxia, anoxia or trauma above what would have happened if all those births had been in weekday working hours; for such births on a weekend/holiday night, there would be one additional neonatal death attributed to asphyxia, anoxia or trauma in every 933 births. This amounts to 46 additional neonatal deaths in England across the 10-year study period, or between 3 and 6 deaths per year (supplementary table 6). It constitutes 18% of the 255 neonatal deaths attributed to asphyxia, anoxia or trauma occurring among emergency caesareans without labour, 3% of the 1,494 neonatal deaths attributed to asphyxia, anoxia or trauma and 0.4% of all neonatal deaths.

Discussion

Statement of principal findings

Overall, this study did not find evidence of a higher risk of all-cause or cause-specific neonatal mortality for spontaneous or instrumental births, or for births by emergency caesarean after spontaneous or induced onset, when born outside of working hours.

Among babies coded as born by emergency caesarean without labour, which accounted for 2% of births in our analysis population, those born at night-time during the week had 56% higher odds of neonatal death attributed to asphyxia, anoxia or trauma when compared with those born during weekday working hours (defined as between 7am and 7pm Monday to Friday); those born at night-time during the weekend or on a holiday had 75% higher odds compared with those born in weekday working hours. In our dataset, with 71,700 births coded as emergency caesareans without labour outside working hours, we estimate that 46 excess deaths are likely to be associated with being born outside of working hours across the ten years.

[31]

Strengths and weaknesses of the study &

Strengths and weaknesses in relation to other studies, discussing important differences in results

Strengths related to the data source

This study used a large, linked dataset which brings together information on birth registration, birth notification and hospital maternity data for over 6 million births over ten years. To date, a cohort of this size has not been used to analyse birth outcomes based on timing in the NHS. The large cohort allowed for stratification by distinct care pathways based on both onset of labour and mode of birth, and for the opportunity to examine mortality attributed to asphyxia, anoxia or trauma, most likely to be affected by care at birth but comprising approximately 11% of neonatal deaths. A study in Scotland found a raised risk of mortality attributed to anoxia in term births [20] but because of the smaller population, the data related to 1,039,560 live births over the 20 years 1985 to 2004 and the numbers were not large enough to stratify by onset of labour and type of birth. The study used data from the Scottish Stillbirth and Infant Death Survey in which information from death certificates was supplemented by further information recorded in clinical settings and classified using a modification

For peer review only - http://bmjopen.bmj.com/site/about/guidelines.xhtml

of the Wigglesworth classification. The definition of anoxia was 'broad, including hypoxia, acidosis and asphyxia'.[20] The ONS modified Wigglesworth classification[26] had been designed to classify conditions recorded on stillbirth and neonatal death certificates and does not use data from other sources. It groups together anoxia, asphyxia and trauma and so the inclusion criteria in our study do not differ significantly from those used in the earlier study.

Moreover, the analysis of Scottish data included only term births whereas our large dataset allowed for adjustment by gestational age. Our findings confirm those of this Scottish report but further identify a specific cohort of births at risk: the subset of births born by emergency caesarean without labour.

The nations of the UK are unusual in stratifying caesarean sections into elective/planned and emergency, while in many other countries the subdivision is into whether they take place before or in labour.[32] The subgroup we have identified would not have been visible if the latter categorisation had been used. An audit of caesarean section in Scotland in 1994/95 found, at a time when the overall caesarean section rate was lower, that 14.1% of caesarean sections were classified as emergency sections before the onset of labour and that these were mainly attributed to fetal growth restriction or fetal distress.[30]

Given the large number of variables available in the linked datasets, we were able to adjust for relevant covariates including obstetric risk factors, unlike the Scottish study. We have also adjusted for the seasonal and temporal nature of births and neonatal mortality over the study period, reported in our previous analyses of this dataset.[22]

Strengths and limitations related to fetal death classification

A study using data from Maternity HES to analyse perinatal mortality by day of the week concluded that mortality was higher at weekends.[33] Without linkage to birth notification, it was unable to take account of the time of day and the authors' analysis and interpretation of their results was highly criticised in subsequent rapid responses.[34-36] Like past analyses of rates of stillbirth and perinatal mortality in England and Wales, this analysis made no distinction between intrapartum and antepartum stillbirths, despite the fact that the majority of stillbirths are antepartum and are therefore most are unlikely to be affected by care at birth.In contrast to other studies, we removed stillbirths from our analyses. To investigate intrapartum stillbirths poses the problem of identifying which stillbirths were definitely intrapartum when for many stillbirths it is unclear whether they occurred before or during labour. One of our advisors, the late Martin Ward Platt, suggested that we obtain confidential enquiry data to supplement the information from stillbirth registration certificates, following the precedent of the Scottish Stillbirth and Infant Death Survey, but it proved impossible to obtain a consistent series of data because of the many changes which took place in the confidential enquiries over the years 2005 to 2014. Permission was obtained from the Healthcare Quality Improvement Partnership to access data compiled by CEMACH and CMACE for births in the years 2005 to 2010, in which there were a number of year-to-year changes.[21] More substantial changes were made from 2012 when responsibility for the confidential enquiry programme passed to Mothers and Babies: Reducing Risk through Audits and Confidential Enquiries across the UK (MBRRACE-UK). Its most recent report found that of the 1939 stillbirths in England in 2020, 89 per cent were antepartum. Of the remainder 141 were classified as occurring intrapartum and 79 being of unknown timing.[37] In view of this continuing difficulty, we did not continue our plans to analyse intrapartum stillbirths.

For peer review only - http://bmjopen.bmj.com/site/about/guidelines.xhtml

Strengths and limitations of classification of caesareans

Our classification of caesareans requires decision-making based on how births are coded, which may not always reflect clinical practice and, particularly for planned caesareans, may be influenced by clinical decisions made at earlier 'booking' antenatal appointments. For births recorded as planned caesareans, we found evidence of an inflated rate ratio for all-cause and cause-specific mortality outside of working hours which may be related to data recording and requires further consideration. Only 29,873 of the 582,990 planned caesareans in our analysis population (5%) took place outside of working hours. Among 'planned caesareans', the underlying population differed according to working hours: planned caesareans usually occur in working hours, and those 'planned caesareans' that occur out of hours are likely to involve complications. An audit of caesarean section in Scotland in 1994/95 found that elective caesareans outside the hours of 9am to 6pm occurred mainly where women were booked for an elective caesarean but went into labour before the planned date.[30] A comparison of OPCS procedure codes and the DELMETH variable from the HES maternity tail published in 2013 found that mode of delivery being recorded as emergency caesarean in one source and planned caesarean in the other was by far the most common type of inconsistency.[31] The scheduling of elective repeat caesareans during working hours is likely to explain the association of previous caesarean birth with birth during working hours as theses have previously been shown to take place primarily on weekday mornings 9–11am.[22] Similarly, higher caesarean rates among older women could explain the higher proportions of births during working hours among older age groups.

We suspect that the crude findings related to planned caesareans was due to misclassification of actual emergency caesareans as 'planned', but there is little way to verify this without booking information and this requires further investigation by other means. There could be an important subgroup of women who are identified as at risk at booking but then progress to become emergencies. The limitations of the administrative data in this study meant we were unable to assess whether this is related to any differences in care in and out of hours.

Additional strengths and limitations

Studies have used 9am to 5pm to represent working hours, but are unlikely to reflect typical working hours in the NHS. We used a wider definition of working hours 7am to 7pm to capture this and to reduce the potential for misclassification.

A key strength is our patient engagement – we have presented the number needed to harm through recommendations from maternity service users who felt that odds ratios and relative risks were not accessible for all to understand and did not fully capture rarity of events and absolute risk. This is also important for planning and strategies. To our knowledge, this is the first time that the NNH has been estimated for neonatal mortality in relation to hospital working hours and birth type.

As with all observational studies, there is a potential for unmeasured confounding despite adjustment for multiple covariates. There is also potential for variation in reporting which can vary between trusts and over time, although the quality and completeness of HES improved over the period covered by this study. Future analyses are needed to confirm if these results persisted after the study period as the introduction in 2015 of a second national system, the Maternity Services Dataset may have competed for staff time available for data recording. Investigations of the 'weekend effect' assume that timing of birth is associated with availability of specialist care, but we did not have any data to directly measure that availability. The size and completeness of our dataset gives us confidence in the accuracy of our findings for NHS births in England as a whole, although variation within England will not be captured in this estimate.

For peer review only - http://bmjopen.bmj.com/site/about/guidelines.xhtml

Meaning of the study: possible explanations and implications for clinicians and policymakers

Overall, out of hours care is not associated with a raised risk of neonatal mortality. Policy should focus on making arrangements for the small subset of emergencies where there may be an association rather than regarding all births out of hours as dangerous. Such arrangements may regard antenatal monitoring of or advice on healthcare-seeking behaviour for parents of particularly vulnerable babies.

Our NNH finds that over a ten-year period there were approximately 46 excess neonatal deaths attributed to asphyxia, anoxia or trauma among babies born out of hours by emergency caesarean without labour.

Emergency caesareans without labour make up approximately 2% of all births over the study period. Compared with other care pathways, they have by far the highest crude rates of neonatal mortality both overall and of mortality attributed to asphyxia, anoxia or trauma, both in and out of working hours.

As such, these births represent a cohort of mothers and babies with high risk, emergent conditions that are, by nature, likely to be unpredictable. The 2017 MBRRACE report on term, singleton, intrapartum stillbirth and intrapartum-related neonatal deaths reported that many of the deaths they reviewed followed pregnancies classified as low-risk.[38] That report also found that in at least a quarter of the 78 cases reviewed, sampled randomly from all eligible deaths, staffing capacity issues played a role in the death. A systematic review of articles on weekend mortality did not establish clear reasons for the higher weekend mortality for general NHS A&E care.[4] That review found no weekend effect in maternity admissions [39] although these were not disaggregated as we have done, and the weekday/weekend difference may elicit different findings that the working/out of hours classification that we used. That study also did not include comparisons of daytime and night-time, which our results suggest may be more important than the weekday/weekend distinction for the small group of births where working hours is associated with higher risk. The present study presents a comparable pattern of increased risk for births that reflect 'A&E-like' emergency maternal care, women not in labour but with conditions which indicate an immediate caesarean. Possible factors of influence might include differences in community healthcare or healthcare-seeking behaviour out of hours, availability of staff out of hours to take calls querying symptoms or to provide procedures such as scans, and transport to maternity units being more difficult out of hours. This could lead to differences in case presentation with maternity users presenting with conditions that require emergency caesareans later than during working hours.

Unanswered questions and future research

We have found that women facing an emergency requiring caesarean birth without labour have a small increased risk of neonatal mortality when giving birth at night. However, the reasons behind this remain unclear, particularly whether the increased risk has its origins in hospital or community care.

Future research should focus on characterising the cohort of women who have an emergency caesarean without labour, whether there are aspects of their care pathways or care-seeking behaviour that would be amenable to intervention, and whether a planned caesarean would have been an option.

For peer review only - http://bmjopen.bmj.com/site/about/guidelines.xhtml

Analyses by NHS trust would not include sufficient numbers of deaths to look at mortality outcomes but could provide useful information on rates of emergency caesareans in and out of hours and ideally could link to information on staff-to-patient ratios. We had hoped to do this in our earlier project using NHS workforce statistics, but found that they were too inconsistent.[21] There may be scope to use the City Birth Cohort for more fine-grained investigation including of variation by trust, if power could be increased using a neonatal near-miss outcome rather than mortality.[40]

License for publication

The Corresponding Author has the right to grant on behalf of all authors and does grant on behalf of all authors, a worldwide licence to the Publishers and its licensees in perpetuity, in all forms, formats and media (whether known now or created in the future), to i) publish, reproduce, distribute, display and store the Contribution, ii) translate the Contribution into other languages, create adaptations, reprints, include within collections and create summaries, extracts and/or, abstracts of the Contribution, iii) create any other derivative work(s) based on the Contribution, iv) to exploit all subsidiary rights in the Contribution, v) the inclusion of electronic links from the Contribution to third party material where-ever it may be located; and, vi) licence any third party to do any or all of the above.

The Submitting Author has the right to grant and does grant on behalf of all authors of the Work an exclusive licence on a worldwide, perpetual, irrevocable, royalty-free basis to BMJ Publishing Group Ltd ("BMJ") and its licensees.

The Submitting Author accepts and understands that any supply made under these terms is made by BMJ to the Submitting Author unless you are acting as an employee on behalf of your employer or a postgraduate student of an affiliated institution which is paying any applicable article publishing charge ("APC") for Open Access articles. Where the Submitting Author wishes to make the Work available on an Open Access basis (and intends to pay the relevant APC), the terms of reuse of such Open Access shall be governed by a Creative Commons licence – details of these licences and which licence will apply to this Work are set out in our licence referred to above.

Transparency statement

The manuscript's guarantor affirms that this manuscript is an honest, accurate, and transparent account of the study being reported; that no important aspects of the study have been omitted; and that any discrepancies from the study as planned have been explained.

Funding

The work described here was funded by the Economic and Social Research Council (ECHR) under its Secondary Data Analysis Initiative. Implications of time of day and day of the week for the outcome of birth' (ES/S010785/1) It draws on earlier work 'Births and their outcomes by time, day and year: a retrospective birth cohort data linkage study', funded by the Health Services and Delivery Research Programme grant 12/136/93. The views expressed are those of the authors and not necessarily those of the ESRC.

Contributor and guarantor information

AM, RP and MCB conceived and designed the analysis and AM is the guarantor of the overall content.

For peer review only - http://bmjopen.bmj.com/site/about/guidelines.xhtml

RP led the patient and public involvement activities.

LC, CG and MCB conducted the analysis.

All authors drafted or gave substantive comments on the manuscript. The corresponding author attests that all listed authors meet authorship criteria and that no others meeting the criteria have been omitted. The authors are grateful to Miranda Scanlon and Dharmintra Pasupathy for comments on the manuscript and to the members of the PI&E group and our project advisory group for their guidance and input.

Competing interests

No competing interests.

Data Sharing Statement

The authors do not have permission to supply data or identifiable information to third parties, including other researchers, but they have permission under Regulation 5 of the Health Service (Control of Patient Information) Regulations 2002 to analyse confidential patient information for England and Wales without consent and create a research database which could be accessed by other researchers using the Secure Research Service at the Office for National Statistics. The information held includes the subset of data and the R computing code used to undertake the analyses described in this article. Anyone wishing to access the linked datasets for research purposes should apply to the Office for National Statistics and NHS England as well as to the Health Research Authority via the Confidentiality Advisory Group to access confidential patient information without consent. In the first instance, enquiries about access to the data should be addressed to the principal investigator, Alison Macfarlane (A.J.Macfarlane@city.ac.uk).

Ethics approval

Ethics approval 05/Q0603/108 and subsequent substantial amendments were granted by East London and City Local Research Ethics Committee 1 and its successors.

Use of patient identifiable data without consent

Permission to use patient identifiable data without consent under Section 60 of the Health and Social Care Act 2001 was initially granted by the Patient Information Advisory Group PIAG 2-10(g)/2005. Renewals and amendments under Section 251 of the Health Service (Control of Patient Information) Regulations 2002 were granted by its successor bodies, the Ethics and Confidentiality Committee of the National Information Governance Board and the Confidentiality Advisory Group of the Health Research Authority.

A second permission CAG 9-08(b)2014 to use patient identifiable data without consent under Section 251 of the Health Service (Control of Patient Information) Regulations 2002 to create a research database held at the Office for National Statistics for analyses relating to inequalities in the outcome of pregnancy and to inform maternity service users about the outcome of midwifery, obstetric and neonatal care was granted by the Confidentiality Advisory Group of the Health Research Authority.

Use of the Office for National Statistics Secure Research Service

Permission to access data from the Office for National Statistics in the VML, now known as the Secure Research Service was granted by ONS's Microdata Release Panel, now superseded by its

For peer review only - http://bmjopen.bmj.com/site/about/guidelines.xhtml

Research Advisory Panel. All members of the research team successfully applied for ONS Approved Researcher Status. The work is covered by the following disclaimer:

'This work was produced using statistical data from ONS. The use of the ONS statistical data in this work does not imply the endorsement of the ONS in relation to the interpretation or analysis of the statistical data. This work uses research datasets which may not exactly reproduce National Statistics aggregates.'

Permission to use data from the Health and Social Care Information Centre.

Permission to link and analyse data held by the Health and Social Care Information Centre, subsequently known as NHS Digital and now part of NHS England, was granted under Data Sharing Agreements NIC-273840-N0N0N and subsequently under DARS-NIC-10094-P6P4B-v6.7 – Linkage, analysis and dissemination of national birth and maternity data for England and Wales

References

1. Bray, B.D. and A. Steventon, *What have we learnt after 15 years of research into the 'weekend effect'?* BMJ Quality & Safety, 2016.
2. Wise, J., *The weekend effect—how strong is the evidence?* BMJ, 2016. **353**.
3. Department of Health, *The Government's mandate to NHS England for 2016-17*. 2015: London.
4. Bion, J., et al., *Increasing specialist intensity at weekends to improve outcomes for patients undergoing emergency hospital admission: the HiSLAC two-phase mixed-methods study*, in *Health Services and Delivery Research*. 2021, NIHR Journals Library: Southampton (UK).
5. Chen, Y.F., et al., *The magnitude and mechanisms of the weekend effect in hospital admissions: A protocol for a mixed methods review incorporating a systematic review and framework synthesis.* Syst Rev, 2016. **5**.
6. Macfarlane, A., *Variations in number of births and perinatal mortality by day of week in England and Wales.* BMJ, 1978. **2**(6153): p. 1670-3.
7. Macfarlane, A., *Day of the week variations in numbers of births and perinatal mortality rates.* Journal of Maternal and Child Health, 1979. **4**: p. 415-416.
8. Macfarlane, A. and P. Thew, *Births: the weekly cycle.* Population Trends, 1978. **13**: p. 23-24.
9. Hawe, E., A. Macfarlane, and J. Bithell, *Daily and seasonal variation in live births, stillbirths and infant mortality in England and Wales, 1979-96.* Health Statistics Quarterly, 2001. **09**: p. 5-15.

For peer review only - http://bmjopen.bmj.com/site/about/guidelines.xhtml

10. Luo, Z.-C., et al., *Risks of stillbirth and early neonatal death by day of week.* Canadian Medical Association Journal, 2004. **170**(3): p. 337-341.

11. Mathers, C.D., *Births and perinatal deaths in Australia: variations by day of week.* J Epidemiol Community Health, 1983. **37**(1): p. 57-62.

12. Palmer, W.L., A. Bottle, and P. Aylin, *Association between day of delivery and obstetric outcomes: observational study.* BMJ, 2015. **351**: p. h5774.

13. Stewart, J.H., J. Andrews, and P.H. Cartlidge, *Numbers of deaths related to intrapartum asphyxia and timing of birth in all Wales perinatal survey, 1993-5.* BMJ, 1998. **316**(7132): p. 657-660.

14. Chalmers, J.W.T., et al., *Scottish data on intrapartum related deaths are in same direction as Welsh data.* BMJ, 1998. **317**(7157): p. 539.

15. Paccaud, F., B. Martin-Beran, and F. Gutzwiller, *Hour of birth as a prognostic factor for perinatal death.* Lancet, 1988. **331**(8581): p. 340-3.

16. Ruffieux, C., A. Marazzi, and F. Paccaud, *The circadian rhythm of the perinatal mortality rate in Switzerland.* American journal of epidemiology, 1992. **135**(8): p. 936-952.

17. Luo, Z.C. and J. Karlberg, *Timing of birth and infant and early neonatal mortality in Sweden 1973-95: longitudinal birth register study.* BMJ, 2001. **323**(7325): p. 1327-30.

18. Stephansson, O., et al., *Time of birth and risk of intrapartum and early neonatal death.* Epidemiology, 2003. **14**(2): p. 218-22.

19. Gould, J.B., C. Qin, and G. Chavez, *Time of birth and the risk of neonatal death.* Obstet Gynecol, 2005. **106**(2): p. 352-8.

20. Pasupathy, D., et al., *Time of birth and risk of neonatal death at term: retrospective cohort study.* BMJ, 2010. **341**: p. c3498.

21. Macfarlane, A., et al., *Births and their outcomes by time, day and year: a retrospective birth cohort data linkage study.* Health Services and Delivery Research, 2019. **7**(18).

22. Martin, P., et al., *Timing of singleton births by onset of labour and mode of birth in NHS maternity units in England, 2005–2014: A study of linked birth registration, birth notification, and hospital episode data.* PLOS ONE, 2018. **13**(6): p. e0198183.

For peer review only

For peer review only - http://bmjopen.bmj.com/site/about/guidelines.xhtml

23. Dattani, N. and A. Macfarlane, *Linkage of Maternity Hospital Episode Statistics data to birth registration and NHS Numbers for births in England 2005–2014: methods. A population-based birth cohort study.* BMJ Open, 2018. **8**: p. e017897.
24. Harper, G., *Linkage of Maternity Hospital Episode Statistics data to birth registration and notification records for births in England 2005–2014: quality assurance of linkage of routine data for singleton and multiple births.* BMJ Open, 2018. **8**: p. e017898.
25. Draper, E., et al., *MBRRACE-UK Perinatal Mortality Surveillance Report, UK Perinatal Deaths for Births from January to December 2020*. 2022, The Infant Mortality and Morbidity Studies, Department of Health Sciences, University of Leicester: Leicester.
26. Dattani, N. and S. Rowan, *Causes of neonatal deaths and stillbirths: a new hierarchical classification in ICD-10.* Health Statistics Quarterly, 2002. **15**.
27. Wigglesworth, J.S., *Monitoring perinatal mortality. A pathophysiological approach.* Lancet, 1980. **2**(8196): p. 684-6.
28. Kuha, J., *AIC and BIC: Comparisons of Assumptions and Performance.* Sociological Methods & Research, 2004. **33**(2): p. 188-229.
29. National Institute for Health Research. *Births and their outcome: analysing the daily, weekly and yearly cyles and their implications for the NHS: Study Documentation*. 2017 15 November 2017]; Available from: https://www.journalslibrary.nihr.ac.uk/programmes/hsdr/1213693/#/.
30. McIlwaine, G., et al., *Caesarean Section in Scotland 1994/5: a National Audit*. 1996.
31. Knight, H.E., et al., *Evaluating maternity care using national administrative health datasets: How are statistics affected by the quality of data on method of delivery?* BMC Health Services Research, 2013. **13**(1): p. 200.
32. Euro-Peristat project with SCPE and Eurocat, *Euro-Peristat Project: European Perinatal Health Report. Core indicators of the health and care of pregnant women and babies in Europe in 2015*. 2018.
33. Aylin, P., et al., *Weekend mortality for emergency admissions. A large, multicentre study.* Quality and Safety in Health Care, 2010. **19**(3): p. 213-217.

For peer review only - http://bmjopen.bmj.com/site/about/guidelines.xhtml

34. Freudenthal, B. *Editorial double-standards for Dr Foster - a major fail for BMJ. BMJ Rapid Response 25 November 2015*. 2015; Available from: http://www.bmj.com/content/351/bmj.h5774/rr-8.
35. Macfarlane, A., et al. *Re: Association between day of delivery and obstetric outcomes: observational study, BMJ Rapid Response 25 November 2015*. 2015; Available from: http://www.bmj.com/content/351/bmj.h5774/rr-7.
36. Richmond, D., et al. *Re: Assocation between day of delivery and obstetric outcomes: observational study. BMJ Rapid Response 25 November 2015*. 2015; Available from: http://www.bmj.com/content/351/bmj.h5774/rr-13.
37. Draper ES, et al., *MBRRACE-UK Perinatal Mortality Surveillance Report, UK Perinatal Deaths for Births from January to December 2020*, in *The Infant Mortality and Morbidity Studies*. 2022, Department of Health Sciences, University of Leicester: Leicester.
38. Draper, E., J. Kurinczuk, and S. Kenyon (eds), *MBBRACE-UK Perinatal Confidential Enquiry: Term, singleton, intrapartum stillbirth and intrapartum-related neonatal death*, MBBRACE-UK, Editor. 2017.
39. Chen, Y.-F., et al., *Magnitude and modifiers of the weekend effect in hospital admissions: a systematic review and meta-analysis.* BMJ Open, 2019. **9**(6): p. e025764.
40. Medeiros, P.d.B., et al., *Neonatal near miss: A review of current definitions and the need for standardisation.* Australian and New Zealand Journal of Obstetrics and Gynaecology, 2022. **62**(3): p. 358-363.

For peer review only - http://bmjopen.bmj.com/site/about/guidelines.xhtml

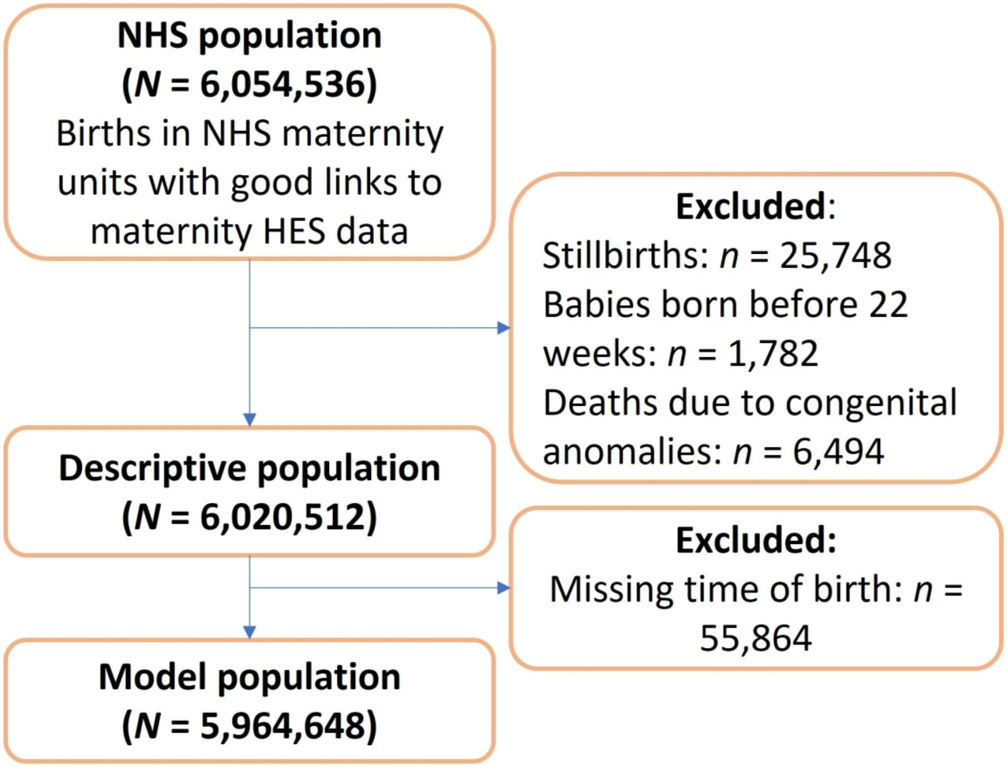

Population

155x120mm (330 x 330 DPI)

Neonatal mortality in NHS maternity units by timing and method of birth: a retrospective linked cohort study

Lucy Carty, Christopher Grollman, Rachel Plachcinski, Mario Cortina-Borja and Alison Macfarlane

[Citation detail]

DOI: [Article DOI]

Supplementary material

Coding of all variables used in the analysis

Coding of variables used in the analysis.

Supplementary table 1. Coding of all variables used in the analysis

**Variable in analysis**	**Original format**	**Coding**
Baby's date of birth	Calendar date	Continuous from first to last day of study
Day in year of baby's date of birth	Calendar date	Continuous from 1 to 365 or 366
Clock go forward/back	Calendar date	Binary for whether clocks went forward/back that day
Working hours (binary)	Time of birth, calendar date	Binary:<br>Working hours = 0700 to 1859 inclusive, Monday to Friday except public holidays<br>Non-working hours = all other times
Working hours (4 categories)	Time of birth, calendar date	Four categories:<br>Working hours (weekday daytime) = 0700 to 1859, Monday to Friday except public holidays<br>Weekday night-time = 1900 to 2359, Monday to Friday except public holidays; 0000 to 0659 Tuesday to Friday except public holidays<br>Weekend/holiday daytime = 0700 to 1859 Saturday, Sunday or public holiday<br>Weekend/holiday night-time = 1900 to 2359, Saturday, Sunday or public holiday; 0000 to 0659 Saturday, Sunday, public holiday, Monday or day after public holiday
Gestational age	Completed weeks since last menstrual period, or by ultrasound	Grouped in categories:<br>22 to 28 weeks; 28 to 32 weeks; 32 to 37 weeks; 37 to 42 weeks; 42 weeks and over; Missing

For peer review only - http://bmjopen.bmj.com/site/about/guidelines.xhtml

Birthweight	Birthweight in grammes	Grouped in categories:<br><br><2500; 2500–2999; 3000–3499; 3500–3999; 4000+
Age of mother	Fractional years	Continuous in completed years
Parity	Number of previous births	Binary nulliparous/multiparous
Ethnicity	17 categories based on the ethnicity question in the 2001 Census of England and Wales	Seventeen categories:<br><br>White British; White Irish; Any other White background; Mixed White & Black Caribbean; Mixed White & Black African; Mixed White & Asian; Any other Mixed background; Asian Indian; Asian Pakistani; Asian Bangladeshi; Any other Asian background; Black Caribbean; Black African; Any other Black background; Chinese; Any other ethnic group; Not Known
Region	Ten Strategic Health Authority regions in the NHS for the majority of the study period	Ten categories: North East; North West; Yorkshire/Humber; East Midlands; West Midlands; East of England; London; South East Coast; South West; South Central
Marital status at birth registration	Categories recorded at birth registration	Four categories:<br><br>Joint registration with different address; Joint same address; Sole registration; Within marriage
Mother's place of birth	Countries recorded at birth registration	Grouped into seven categories:<br><br>United Kingdom; Other Europe; Africa; The Americas and the Caribbean; Middle East and Asia; Oceania and Antarctica; Missing
Previous caesarean birth	Whether present (yes/no)	Binary no/any previous caesarean birth
Obstetric complications*	Whether present (yes/no)	Binary not present/present

For peer review only

* Hypertension, Preeclampsia, Eclampsia, Diabetes, Gestational diabetes, Malpresentation of foetus, Maternal care for disproportion, Maternal care for abnormality of pelvic organs, Polyhydramnios, Oligohydramnios, Premature rupture of membranes, Placental disorders, Placenta praevia, Premature separation of placenta, Antepartum haemorrhage, Obstructed labour, Intrapartum haemorrhage, Umbilical cord complications

Crude all-cause neonatal mortality rates

Crude all-cause neonatal mortality rates in the analysis population were higher during non-working hours compared with working hours (1.58 vs 1.36 per 1000 live births, rate ratio 1.17, 95% confidence interval 1.12 to 1.22) (Supplementary table 2). The crude mortality rate ratio was not significantly different from 1 for spontaneous or instrumental births and was below 1 for emergency caesarean births (0.86, 0.80 to 0.93), meaning lower crude all-cause neonatal mortality rate during non-working hours compared with working hours (3.13 vs 3.62 per 1000 live births).

For peer review only - http://bmjopen.bmj.com/site/about/guidelines.xhtml

Supplementary table 2. Crude all-cause neonatal mortality by time and day of birth, stratified by method of birth

	Living at 28 days	Neonatal death	Total	Rate*	Rate ratio	95% CI
**Total population**						
Working hours	2325569	3162	2328731	1.36		
Non-working hours	3630159	5758	3635917	1.58	1.17	1.12 to 1.22
**Spontaneous delivery**						
Working hours	1201063	1647	1202710	1.37		
Non-working hours	2554815	3542	2558357	1.38	1.01	0.95 to 1.07
**Instrumental delivery**						
Working hours	256279	173	256452	0.67		
Non-working hours	485085	340	485425	0.70	1.04	0.87 to 1.25
**Emergency caesarean**						
Working hours	310092	1126	311218	3.62		
Non-working hours	553239	1735	554974	3.13	0.86	0.8 to 0.93
**Planned caesarean**						
Working hours	552917	200	553117	0.36		
Non-working hours	29791	82	29873	2.74	7.61	5.86 to 9.8

* Per 1000 live births

Adjusted odds of all-cause neonatal mortality by method of birth

After adjustment, the 'protective' effect of birth in non-working hours for emergency caesareans, seen in the crude rates, was no longer evident. Odds ratios for spontaneous, instrumental and emergency caesarean deliveries were not significantly different from 1 (Supplementary table 3).

Supplementary table 3. Unadjusted and adjusted odds ratios for all-cause neonatal mortality during non-working hours versus working hours by delivery type

	Odds ratio for neonatal mortality during non-working hours compared with working hours			
	Unadjusted OR	95% CI	Adjusted OR*	95% CI
**All-cause neonatal mortality**				
Spontaneous delivery	1.01	0.95 to 1.07	0.99	0.93 to 1.06
Instrumental delivery	1.04	0.87 to 1.25	1.11	0.91 to 1.34
Emergency caesarean	0.86	0.80 to 0.93	1.06	0.98 to 1.14

* Adjusted for baby's sex, gestational age, birthweight, mode of onset of labour, geographical region, NHS trust, baby's ethnicity, baby's date of birth, maternal age and maternal parity, as well as yearly harmonic terms for day of year of the birth.

For peer review only - http://bmjopen.bmj.com/site/about/guidelines.xhtml

Obstetric risk factor characteristics for emergency caesareans without labour

We compared obstetric risk factor characteristics for emergency caesareans without labour during working and non-working hours (supplementary materials) (Supplementary table 4).

Supplementary table 4. Obstetric characteristics and risk factors for emergency caesareans without labour by working and non-working hours

Characteristics and risk factors	Level	Working hours	%	Non-working hours	%	*p*-value	SMD
N		60,202		71,700			
Baby's sex	F	28,505	47.3	33,670	47.0	0.160	0.008
	M	31,697	52.7	38,030	53.0		
Gestational age	22 to 28 weeks	1,127	1.9	1,439	2.0	<0.001	0.034
	28 to 32 weeks	4,182	6.9	4,926	6.9		
	32 to 37 weeks	14,368	23.9	17,167	23.9		
	37 to 42 weeks	38,228	63.5	45,008	62.8		
	42+ weeks	2,062	3.4	2,880	4.0		
	Missing	235	0.4	280	0.4		
Birthweight	Under 2,500	16,653	27.7	20,629	28.8	<0.001	0.054
	2,500-2,999	11,214	18.6	14,367	20.0		
	3,000-3,499	15,229	25.3	17,638	24.6		
	3,500-3,999	10,759	17.9	12,083	16.9		
	4,000 and over	5,156	8.6	5,691	7.9		
	Missing or unfeasible	1,191	2.0	1,292	1.8		
Maternal age	Under 20	1,899	3.2	2,697	3.8	<0.001	0.060
	20-24	8,229	13.7	10,529	14.7		
	25-29	14,603	24.3	18,051	25.2		
	30-34	18,359	30.5	21,420	29.9		
	35-39	12,920	21.5	14,444	20.1		
	Over 40	4,192	7.0	4,559	6.4		
Parity	Nulliparous	22,316	37.1	29,730	41.5	<0.001	0.091
	Moderate: 1 to 4	35,249	8.6	38,885	54.2		
	High: 5 to 9	2,511	4.2	2,940	4.1		
	Very high: 10 to 14	111	0.2	132	0.2		
	Extreme, unfeasible or missing	X	0.0	X	0.0		
Ethnicity	White British	35,770	59.4	41,519	57.9	<0.001	0.041
	White Irish	427	0.7	537	0.7		
	Any other White background	3,946	6.6	4,714	6.6		
	Mixed White & Black Caribbean	581	1.0	765	1.1		

For peer review only - http://bmjopen.bmj.com/site/about/guidelines.xhtml

	Mixed White & Black African	427	0.7	491	0.7		
	Mixed White & Asian	670	1.1	814	1.1		
	Any other Mixed background	1,064	1.8	1,365	1.9		
	Asian Indian	2,349	3.9	3,056	4.3		
	Asian Pakistani	2,910	4.8	3,706	5.2		
	Asian Bangladeshi	1,157	1.9	1,500	2.1		
	Any other Asian background	1,171	1.9	1,352	1.9		
	Black Caribbean	772	1.3	991	1.4		
	Black African	3,377	5.6	4,262	5.9		
	Any other Black background	609	1.0	737	1.0		
	Chinese	239	0.4	275	0.4		
	Any other ethnic group	1,378	2.3	1,698	2.4		
	Not Known	3,355	5.6	3,918	5.5		
Region	North East	2,053	3.4	2,405	3.4	<0.001	0.080
	North West	10,040	16.7	12,628	17.6		
	Yorkshire/Humber	4,509	7.5	5,325	7.4		
	East Midlands	2,974	4.9	3,082	4.3		
	West Midlands	8,753	14.5	12,053	16.8		
	East of England	6,423	10.7	7,572	10.6		
	London	12,590	20.9	14,129	19.7		
	South East Coast	4,254	7.1	5,047	7.0		
	South West	3,589	6	4,059	5.7		
	South Central	5,017	8.3	5,400	7.5		
Marital status at birth registration	Joint registration different address	5,524	9.2	7,082	9.9	<0.001	0.045
	Joint registration same address	16,280	27	20,222	28.2		
	Sole registration	3,749	6.2	4,702	6.6		
	Within marriage	34,649	57.6	39,694	55.4		
Mother's place of birth	United Kingdom	42,735	71	50,128	69.9	<0.001	0.028
	Other Europe	4,070	6.8	4,997	7.0		
	Africa	5,022	8.3	6,242	8.7		
	The Americas and the Caribbean	1,096	1.8	1,258	1.8		
	Asia and Middle East	6,955	11.6	8,733	12.2		
	Oceania, Antarctica and other	298	0.5	304	0.4		
	Missing	26	0.0	38	0.1		
Previous caesarean	Yes	19,030	31.6	18,119	25.3	<0.001	0.141

For peer review only - http://bmjopen.bmj.com/site/about/guidelines.xhtml

Hypertension	Yes	942	1.6	956	1.3	<0.001	0.019
Preeclampsia	Yes	6,902	11.5	8,391	11.7	0.181	0.007
Eclampsia	Yes	294	0.5	352	0.5	0.978	<0.001
Diabetes	Yes	1,600	2.7	1,463	2.0	<0.001	0.041
Gestational diabetes	Yes	3,333	5.5	3,401	4.7	<0.001	0.036
Malpresentation of foetus	Yes	9,605	16	10,639	14.8	<0.001	0.031
Maternal care for disproportion	Yes	104	0.2	155	0.2	0.087	0.010
Maternal care for abnormality of pelvic organs	Yes	17,269	28.7	16,217	22.6	<0.001	0.139
Polyhydramnios	Yes	1,301	2.2	1,377	1.9	0.002	0.017
Oligohydramnios	Yes	2,014	3.3	1,973	2.8	<0.001	0.035
Premature rupture of membranes	Yes	5,874	9.8	8,560	11.9	<0.001	0.070
Placental disorders	Yes	1,375	2.3	1,577	2.2	0.310	0.006
Placenta praevia	Yes	3,374	5.6	3,436	4.8	<0.001	0.037
Premature separation of placenta	Yes	1,831	3	3,305	4.6	<0.001	0.082
Antepartum haemorrhage	Yes	2,178	3.6	3,327	4.6	<0.001	0.051
Obstructed labour	Yes	3,280	5.4	4,502	6.3	<0.001	0.035
Intrapartum haemorrhage	Yes	410	0.7	570	0.8	0.018	0.013
Umbilical cord complications	Yes	1,166	1.9	1,864	2.6	<0.001	0.045

SMD = standardised mean difference; X = suppressed due to count(s) below 10

Point estimates from final fully adjusted model

Supplementary table 5. Odds ratios for neonatal mortality attributed to anoxia, asphyxia or trauma among emergency caesarean births without labour from 2005 to 2014

	Odds ratio	95% CI lower	95% CI upper
Spline of baby's date of birth	1.415	0.586	3.416
Spline of baby's date of birth squared	0.611	0.386	0.967
Sine term related to day-in-year	0.994	0.833	1.185
Cosine term related to day-in-year	1.031	0.867	1.226
Clocks go forward*	0	NA	NA
Clocks go back*	0	NA	NA
Weekday daytime	Ref		
Weekday night-time	1.558	1.152	2.106
Weekend/holiday daytime	0.954	0.626	1.456
Weekend/holiday night-time	1.753	1.242	2.473

For peer review only

For peer review only - http://bmjopen.bmj.com/site/about/guidelines.xhtml

Gestational age 22 to 28 weeks	0.608	0.212	1.747
Gestational age 28 to 32 weeks	0.775	0.417	1.440
Gestational age 32 to 37 weeks	1.109	0.773	1.590
Gestational age 37 to 42 weeks	Ref		
Gestational age 42 weeks and over	0.348	0.128	0.948
Gestational age missing	0.825	0.113	6.034
Birth weight under 2,500	0.981	0.623	1.545
Birth weight 2,500 to 2,999	1.367	0.931	2.008
Birth weight 3,000 to 3,499	Ref		
Birth weight 3,500 to 3,999	1.136	0.744	1.733
Birth weight 4,000 and over	1.353	0.809	2.261
Birth weight missing or unfeasible	2.774	1.395	5.517
North East	1.349	0.613	2.967
North West	1.090	0.658	1.807
Yorkshire/Humber	2.132	1.244	3.653
East Midlands	1.812	0.884	3.716
West Midlands	1.343	0.804	2.244
East of England	1.322	0.761	2.295
London	Ref		
South East Coast	1.121	0.577	2.178
South Central	1.700	0.967	2.990
South West	1.619	0.880	2.978
Spline of age of mother	0.536	0.161	1.787
Spline of age of mother squared	0.175	0.022	1.371
Nulliparous	1.184	0.906	1.547
Multiparous	Ref		
Parity missing*	0	NA	NA
Placental abruption	4.526	3.279	6.248
Maternal treatment for pelvic abnormality	0.357	0.229	0.558
Malpresentation	0.279	0.155	0.500
Preeclampsia	0.306	0.160	0.585
Postpartum haemorrhage	1.704	1.287	2.257
Antepartum haemorrhage	2.282	1.547	3.367

* No deaths; Ref = reference category; CI = confidence interval

Neonatal deaths attributed to anoxia, asphyxia or trauma by year

The number of births each year by emergency caesarean without labour rose from 2005 to 2014, with a larger rise in births during non-working hours than in working hours. Applying the number needed to harm of 1258 to those births during weeknights and 933 to those births during weekend/holiday nights gives a number of annual deaths potentially associated with birth during non-working hours ranging from 3.2 deaths in 2005 to 6.2 deaths in 2014 (Supplementary table 6).

Supplementary table 6. Number of deaths per year attributable to anoxia, asphyxia or trauma potentially associated with birth during non-working hours, among emergency caesarean deliveries without labour

For peer review only - http://bmjopen.bmj.com/site/about/guidelines.xhtml

Birth year	Weekday daytime N births	Weekday night-time (NNH = 1258) N births	Excess deaths**	*Weekend/holiday daytime (NNH = 15,373)** *N births*	*Excess deaths*	Weekend/holiday night-time (NNH = 933) N births	Excess deaths	Total excess deaths
2005	4416	2317	1.8	*1435*	*0.1*	1252	1.3	3.2
2006	4650	2448	1.9	*1426*	*0.1*	1273	1.4	3.3
2007	4623	2378	1.9	*1382*	*0.1*	1223	1.3	3.2
2008	4716	2493	2.0	*1535*	*0.1*	1277	1.4	3.4
2009	6020	3505	2.8	*2020*	*0.1*	1873	2.0	4.8
2010	6450	3610	2.9	*2087*	*0.1*	1931	2.1	4.9
2011	6855	3920	3.1	*2385*	*0.2*	2159	2.3	5.4
2012	7336	4204	3.3	*2581*	*0.2*	2324	2.5	5.8
2013	7455	4200	3.3	*2509*	*0.2*	2330	2.5	5.8
2014	7681	4483	3.6	*2708*	*0.2*	2432	2.6	6.2
Total			26.7		NA		19.4	46.0

* Not included in total as the association of birth during weekend and holiday daytimes with neonatal mortality attributed to anoxia, asphyxia or trauma was not significant

** N births / NNH (number needed to harm)

For peer review only

The RECORD statement – checklist of items, extended from the STROBE statement, that should be reported in observational studies using routinely collected health data.

	Item No.	STROBE items	Location in manuscript where items are reported	RECORD items	Location in manuscript where items are reported
**Title and abstract**					
	1	(a) Indicate the study's design with a commonly used term in the title or the abstract (b) Provide in the abstract an informative and balanced summary of what was done and what was found	Title and abstract	RECORD 1.1: The type of data used should be specified in the title or abstract. When possible, the name of the databases used should be included.<br><br>RECORD 1.2: If applicable, the geographic region and timeframe within which the study took place should be reported in the title or abstract.<br><br>RECORD 1.3: If linkage between databases was conducted for the study, this should be clearly stated in the title or abstract.	1.1 Abstract, manuscript p2: "Retrospective cohort linking birth registration, birth notification and hospital episode data"<br><br>1.2 Abstract, p2: "liveborn singleton births from 2005 to 2014 in NHS maternity units in England"<br><br>1.3 Abstract, p2: "Retrospective cohort linking birth registration, birth notification and hospital episode data"
**Introduction**					
Background rationale	2	Explain the scientific background and rationale for the investigation being reported	Introduction, p2-3		
Objectives	3	State specific objectives, including any prespecified hypotheses	Introduction, p2-3.		
**Methods**					
Study Design	4	Present key elements of study design early in the paper	Methods, p3-5: Data, Selection of Data for Analysis, and Statistical Analysis		

For peer review only

Setting	5	Describe the setting, locations, and relevant dates, including periods of recruitment, exposure, follow-up, and data collection	Methods, p3-4: Selection of Data for Analysis, Data		
Participants	6	*(a) Cohort study* - Give the eligibility criteria, and the sources and methods of selection of participants. Describe methods of follow-up<br>*Case-control study* - Give the eligibility criteria, and the sources and methods of case ascertainment and control selection. Give the rationale for the choice of cases and controls<br>*Cross-sectional study* - Give the eligibility criteria, and the sources and methods of selection of participants<br><br>*(b) Cohort study* - For matched studies, give matching criteria and number of exposed and unexposed<br>*Case-control study* - For matched studies, give matching criteria and the number of controls per case	Methods, p3 & fig: Data and Figure 1	RECORD 6.1: The methods of study population selection (such as codes or algorithms used to identify subjects) should be listed in detail. If this is not possible, an explanation should be provided.<br><br>RECORD 6.2: Any validation studies of the codes or algorithms used to select the population should be referenced. If validation was conducted for this study and not published elsewhere, detailed methods and results should be provided.<br><br>RECORD 6.3: If the study involved linkage of databases, consider use of a flow diagram or other graphical display to demonstrate the data linkage process, including the number of individuals with linked data at each stage.	6.1 Methods, p3: Selection of Data for Analysis, Data<br><br>6.2 References (p19-21) to Macfarlane et al 2019). Martin et al (2018), Dattani et al (2018) and Harper et al (2018)<br><br>6.3 No linkage in this study itself, and linkage well demonstrated in references cited in 6.2 above (p19-21).
Variables	7	Clearly define all outcomes, exposures, predictors, potential confounders, and effect modifiers. Give diagnostic criteria, if applicable.		RECORD 7.1: A complete list of codes and algorithms used to classify exposures, outcomes, confounders, and effect modifiers should be provided. If these cannot be reported, an explanation should be provided.	Supplementary material, p1-2
Data sources/ measurement	8	For each variable of interest, give sources of data and details of methods of assessment (measurement).	Methods, p3-5: Statistical Analysis		

For peer review only

		Describe comparability of assessment methods if there is more than one group			
Bias	9	Describe any efforts to address potential sources of bias	Methods, p3-5: Statistical analysis		
Study size	10	Explain how the study size was arrived at	Methods, p3: Selection of data for analysis, Fig. 1		
Quantitative variables	11	Explain how quantitative variables were handled in the analyses. If applicable, describe which groupings were chosen, and why	Methods, p3-5: Statistical Analysis		
Statistical methods	12	(a) Describe all statistical methods, including those used to control for confounding<br>(b) Describe any methods used to examine subgroups and interactions<br>(c) Explain how missing data were addressed<br>(d) *Cohort study* - If applicable, explain how loss to follow-up was addressed<br>*Case-control study* - If applicable, explain how matching of cases and controls was addressed<br>*Cross-sectional study* - If applicable, describe analytical methods taking account of sampling strategy<br>(e) Describe any sensitivity analyses	Methods, p3-5: Statistical Analysis		
Data access and cleaning methods		..		RECORD 12.1: Authors should describe the extent to which the investigators had access to the database population used to create the study population.	12.1 Methods, p3: Data

For peer review only

				RECORD 12.2: Authors should provide information on the data cleaning methods used in the study.	12.2 This issue was addressed at an earlier stage of the project, see DOIs 10.3310/hsdr07180 and 10.1136/bmjopen-2017-017898
Linkage		..		RECORD 12.3: State whether the study included person-level, institutional-level, or other data linkage across two or more databases. The methods of linkage and methods of linkage quality evaluation should be provided.	12.3 Methods, p3: Data
**Results**					
Participants	13	(a) Report the numbers of individuals at each stage of the study (*e.g.*, numbers potentially eligible, examined for eligibility, confirmed eligible, included in the study, completing follow-up, and analysed)<br>(b) Give reasons for non-participation at each stage.<br>(c) Consider use of a flow diagram	Methods: Data and Figure 1	RECORD 13.1: Describe in detail the selection of the persons included in the study (*i.e.,* study population selection) including filtering based on data quality, data availability and linkage. The selection of included persons can be described in the text and/or by means of the study flow diagram.	Methods, p3: Selection of data for analysis and Figure 1
Descriptive data	14	(a) Give characteristics of study participants (*e.g.*, demographic, clinical, social) and information on exposures and potential confounders<br>(b) Indicate the number of participants with missing data for each variable of interest<br>(c) *Cohort study* - summarise follow-up time (*e.g.*, average and total amount)	Results: Table 2 (p8); Online supplement table 4 (p4-6)		

For peer review only

Outcome data	15	*Cohort study* - Report numbers of outcome events or summary measures over time<br>*Case-control study* - Report numbers in each exposure category, or summary measures of exposure<br>*Cross-sectional study* - Report numbers of outcome events or summary measures	Results, table 3 (p10)		
Main results	16	(a) Give unadjusted estimates and, if applicable, confounder-adjusted estimates and their precision (e.g., 95% confidence interval). Make clear which confounders were adjusted for and why they were included<br>(b) Report category boundaries when continuous variables were categorized<br>(c) If relevant, consider translating estimates of relative risk into absolute risk for a meaningful time period	Results: Tables 3-8 and section Absolute risks from emergency caesarean without labour during non-working hours (pp10-13)		
Other analyses	17	Report other analyses done—e.g., analyses of subgroups and interactions, and sensitivity analyses	Results, p12: Timing within non-working hours		
**Discussion**					
Key results	18	Summarise key results with reference to study objectives	Discussion, p13-14		
Limitations	19	Discuss limitations of the study, taking into account sources of potential bias or imprecision. Discuss both direction and magnitude of any potential bias	Discussion, p15-16	RECORD 19.1: Discuss the implications of using data that were not created or collected to answer the specific research question(s). Include discussion of misclassification bias, unmeasured confounding, missing data, and changing eligibility over	Discussion, p15-16

For peer review only

				time, as they pertain to the study being reported.	
Interpretation	20	Give a cautious overall interpretation of results considering objectives, limitations, multiplicity of analyses, results from similar studies, and other relevant evidence	Discussion, p16		
Generalisability	21	Discuss the generalisability (external validity) of the study results	Discussion, p15		
**Other Information**					
Funding	22	Give the source of funding and the role of the funders for the present study and, if applicable, for the original study on which the present article is based	Funding statement, p19		
Accessibility of protocol, raw data, and programming code		..		RECORD 22.1: Authors should provide information on how to access any supplemental information such as the study protocol, raw data, or programming code.	Supplementary information is provided in an online supplement. Complete open data sharing is not possible because of restriction of access to personal data used in this study. Manuscript p18

*Reference: Benchimol EI, Smeeth L, Guttmann A, Harron K, Moher D, Petersen I, Sørensen HT, von Elm E, Langan SM, the RECORD Working Committee. The REporting of studies Conducted using Observational Routinely-collected health Data (RECORD) Statement. *PLoS Medicine* 2015; in press.

*Checklist is protected under Creative Commons Attribution (CC BY) license.

For peer review only - http://bmjopen.bmj.com/site/about/guidelines.xhtml
